# Genetic and demographic signatures accompanying the evolution of the selfing syndrome in *Daphne kiusiana*, an evergreen shrub

**DOI:** 10.1093/aob/mcac142

**Published:** 2022-12-05

**Authors:** Eun-Kyeong Han, Ichiro Tamaki, Sang-Hun Oh, Jong-Soo Park, Won-Bum Cho, Dong-Pil Jin, Bo-Yun Kim, Sungyu Yang, Dong Chan Son, Hyeok-Jae Choi, Amarsanaa Gantsetseg, Yuji Isagi, Jung-Hyun Lee

**Affiliations:** Department of Biology Education, Chonnam National University, Gwangju 61186, Republic of Korea; Gifu Academy of Forest Science and Culture, 88 Sodai, Mino, Gifu 501-3714, Japan; Department of Biology, Daejeon University, Daejeon 34520, Republic of Korea; Department of Botany, Honam National Institute of Biological Resources, Mokpo 58762, Republic of Korea; Department of Plant Variety Protection, National Forest Seed and Variety Center, Chungju 27495, Republic of Korea; Urban Biodiversity Research Division, Sejong National Arboretum, Sejong 30106, Republic of Korea; Plant Resources Division, National Institute of Biological Resources, Incheon 22689, Republic of Korea; Herbal Medicine Resources Research Center, Korea Institute of Oriental Medicine, Naju 58245, Republic of Korea; Division of Forest Biodiversity and Herbarium, Korea National Arboretum, Pocheon 11186, Republic of Korea; Department of Biology and Chemistry, Changwon National University, Changwon 51140, Republic of Korea; Department of Biology Education, Chonnam National University, Gwangju 61186, Republic of Korea; Division of Forest and Biomaterials Science, Graduate School of Agriculture, Kyoto University, Kyoto 606-8502, Japan; Department of Biology Education, Chonnam National University, Gwangju 61186, Republic of Korea

**Keywords:** *Daphne kiusiana*, demographic dynamics, directional selection, evergreen shrub, inflorescence, NGS technique, selfing syndrome

## Abstract

**Background and Aims:**

The evolution of mating systems from outcrossing to self-fertilization is a common transition in flowering plants. This shift is often associated with the ‘selfing syndrome’, which is characterized by less visible flowers with functional changes to control outcrossing. In most cases, the evolutionary history and demographic dynamics underlying the evolution of the selfing syndrome remain poorly understood.

**Methods:**

Here, we characterize differences in the demographic genetic consequences and associated floral-specific traits between two distinct geographical groups of a wild shrub, *Daphne kiusiana*, endemic to East Asia; plants in the eastern region (southeastern Korea and Kyushu, Japan) exhibit smaller and fewer flowers compared to those of plants in the western region (southwestern Korea). Genetic analyses were conducted using nuclear microsatellites and chloroplast DNA (multiplexed phylogenetic marker sequencing) datasets.

**Key Results:**

A high selfing rate with significantly increased homozygosity characterized the eastern lineage, associated with lower levels of visibility and herkogamy in the floral traits. The two lineages harboured independent phylogeographical histories. In contrast to the western lineage, the eastern lineage showed a gradual reduction in the effective population size with no signs of a severe bottleneck despite its extreme range contraction during the last glacial period.

**Conclusions:**

Our results suggest that the selfing-associated morphological changes in *D. kiusiana* are of relatively old origin (at least 100 000 years ago) and were driven by directional selection for efficient self-pollination. We provide evidence that the evolution of the selfing syndrome in *D. kiusiana* is not strongly associated with a severe population bottleneck.

## INTRODUCTION

Most flowering plants have perfect flowers in where the anthers and stigmas of a single sporophyte are held in close spatiotemporal proximity (Busch and Delph, 2012). Although seemingly ordinary, these arrangements allow for a flexible mating system [outcrossing, self-fertilization (hereafter ‘selfing’] or a combination of both) that can induce strategic decisions to develop offspring in response to variations in environmental factors (Lloyd, 1979; Barrett and Eckert, 1990; Pannell and Barrett, 1998). Selfing has advantages that may be favourable in two circumstances. First, selfing can provide reproductive assurance when pollinators and/or potential mates are limited (Darwin, 1876; Eckert *et al*., 2006). Second, plants that undergo selfing also harbour improved colonization ability, which can act when migrating to a new habitat (Baker, 1955; Foxe *et al*., 2009). In general, selfing is considered a mating strategy that can be substituted for outcrossing when its benefits outweigh the costs of inbreeding depression (Lloyd, 1979, 1992; Busch and Delph, 2012).

The selfing species/lineages derived from outcrossing ancestors are often accompanied by a common set of morphological and functional changes to the flowers, termed the ‘selfing syndrome’ (Ornduff, 1969; Sicard *et al*., 2011). Compared with their outcrossing sister taxa, selfers generally tend to have smaller flowers that narrow the spatial separation between anthers and stigmas, which appears to be effective for self-pollination (Takebayashi *et al*., 2006; Slotte *et al*., 2012). Consequently, a mating system shift toward selfing has occurred independently in many plants (Stebbins, 1974; Grant, 1981; Barrett, 2002), and it has been followed by very similar changes in floral morphology and function (Woźniak *et al*., 2020). It is therefore conceivable that the concerted flower changes for a given species/lineage have been driven by persistent natural selection, even if such advantages are only in the short term (Sicard *et al*., 2011, 2016; Slotte *et al*., 2012; Woźniak *et al*., 2020). Such floral characteristics are thought to partly reflect diverse ecological scenarios that have driven the evolution of the selfing lineage (Runions and Geber, 2000). Nevertheless, our knowledge of the historical factors leading to these morphological changes remains limited. A better understanding of the evolution of the selfing syndrome requires insight into the population establishment histories associated with Quaternary climatic oscillations, which have played a major role in changing the geographical distribution of plant species (Comes and Kadereit, 1998; Qiu *et al*., 2011).

A few population-scale genetic analyses have been informative regarding the demographic histories associated with the evolution of the selfing syndrome. Studies have suggested that the transition to selfing of *Capsella rubella* was driven preliminarily by a severe population bottleneck within the last 50 000 years (Foxe *et al.*, 2009; Guo *et al.*, 2009). Subsequently, its morphological modifications are thought to have evolved relatively rapidly during the geographical expansion after the Last Glacial Maximum (Foxe *et al*., 2009; Guo *et al.*, 2009). These inferences can lead to the perception that the selfing syndrome is not adaptive since it is associated with random genetic drift on a relatively short evolutionary timescale. However, a strong genetic drift can occur regardless of the evolution of mating system or after such flower changes in selfing lineages (Sicard *et al*., 2011). The relationship between the evolution of the selfing syndrome and historical demographic change remains ambiguous. To gain keen insights, it is necessary to account for the demographic details of more species associated with evolution of the selfing syndrome.

**Fig. 1.**
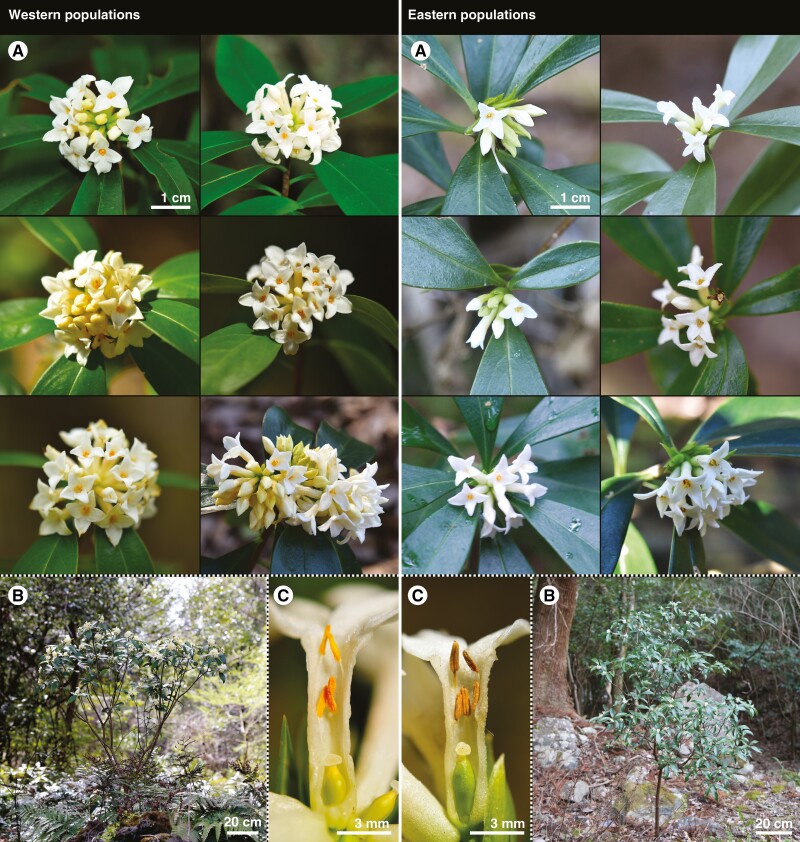
Comparison of floral morphological characteristics according to the geographical division of *Daphne kiusiana*. The western populations show more and larger flowers, whereas the eastern populations show fewer and smaller flowers. The longitudinal sections of the flowers illustrate that the levels of herkogamy are significantly different between the western and eastern populations. (A) inflorescences; (B) habitat; (C) longitudinal section of the flower.

*Daphne kiusiana* is an evergreen broad-leaved shrub endemic to East Asia (Murata, 1999; Lee, 2003; Wang *et al*., 2007) that has perfect flowers and is capable of spontaneous selfing (Huang, 2017). Two varieties of this species have been recognized – var. *kiusiana* (distributed in Korea and Japan) and var. *atrocaulis* (found in China and Taiwan). The former has small flowers with calyx tubes 7–8 mm long, whereas the latter has slightly larger flowers with calyx tubes 10–14 mm long (Murata, 1999; Lee, 2003; Wang *et al*., 2007). However, within var. *kiusiana*, the floral sizes (calyx tubes 12–22 mm long) in the populations on offshore islands of southwestern Korea (hereafter referred to as western populations) are much larger than those in southeastern Korea and Kyushu, Japan (referred to as eastern populations) (Lee *et al*., 2013*a*; Lee and Oh, 2017). These morphological patterns are typical for selfing lineages and their outcrossing ancestors. Indeed, in addition to the characteristics of the selfing syndrome mentioned above, our field observations show that plants in the western populations have many more flowers per inflorescence than those in the eastern populations, with 5–12 flowers per inflorescence (Fig. 1).

Selfing leads to increased homozygosity through the fixation of alleles at gene loci and reduces effective recombination (Nordborg and Donnelly, 1997; Yang *et al*., 2009; Manzanares *et al*., 2016). Therefore, we expected to observe a loss of genetic diversity (Wright *et al*., 2013), an increase in genetic differentiation (Duminil *et al.*, 2009; Voillemot and Pannell, 2017) and differences in the floral traits within the eastern populations (with smaller flowers) compared to the western populations. Genotyping using microsatellite markers offers an ideal technique for testing these expectations, especially when estimating selfing rates (Voillemot and Pannell, 2017; Zhong *et al*., 2019; Gasca-Pineda *et al*., 2020) and lineage divergence times (Tamaki *et al*., 2019). In addition, maternally inherited chloroplast DNA (cpDNA) reflects past seed gene flow (Ennos *et al*., 1999; Calviño-Cancela *et al*., 2012). Therefore, using cpDNA as an auxiliary tool, it is possible to address the historical geographical influence of distribution changes. With advances in next-generation sequencing technology, it is now possible to produce large datasets cost-effectively at the population level. Recently, a multiplexed phylogenetic marker sequencing (MPM-seq) technique for large data generation has been developed to obtain a good understanding of plastid genetic information (Suyama *et al*., 2021). Therefore, here we produced the chloroplast sequences using the MPM-seq method with minor modification and provide the detailed methods.

To our knowledge, the majority of previous population-level studies related to the evolution of the selfing syndrome have been of herbaceous plants. In this study, we aim to elucidate the evolutionary history of a wild shrub, *D. kiusiana*, with subcapitate inflorescences focusing on genetic characteristics, palaeodistribution, historical population demography and floral morphological traits between the two geographical populations (west vs. east). We address the following specific questions: (1) What differences do the genetic makeups of these two geographical groups exhibit? (2) What factors promoted the evolution of the eastern populations with inconspicuous (visually impoverished) floral traits that have probably been induced by selfing? (3) How are the demographic signatures of the group with these characteristic flowers exhibited? We characterize the genetic composition of *D. kiusiana* distributed in Korea and Japan using nuclear microsatellites and multilocus-based cpDNA datasets, and examine the associated variations in morphological floral traits. Finally, we employ ecological niche modelling (ENM) and approximate Bayesian computation (ABC) to gain further insights into the historical migration patterns and demographic details of these populations. Our study provides evolutionary insights into how selfing affects the sustainability and adaptive potential of wild plant populations.

## MATERIALS AND METHODS

### Study species and population sampling

*Daphne kiusiana* Miq. is commonly used as an ornamental plant because of its graceful inflorescence shape (resembling a bride’s bouquet) and pleasant fragrance (Ro *et al*., 2010). In Korea, the populations are now extremely restricted to a few islands, and the plants are being managed as an endangered species (Korea National Arboretum, 2008). In comparison, Japanese plants are widely distributed throughout the southern region. Flowering in Korea and Japan occurs from winter to early spring between January and April (Murata, 1999; Lee, 2003). Approximately 90 d after fertilization, the berries mature, and the seeds are then dispersed by birds (Aoki *et al*., 2004). In garden conditions, seed production usually commences when the plants are 5–6 years old (S. H. Yang, Halla Arboretum, Republic of Korea; pers. comm.). However, because most wild seeds have a dormancy of several years before germination (Alonso and Herrera, 2001; Fang *et al*., 2016), the life cycle of *D. kiusiana* is thought to span a period of ~10 years.

Our study was conducted on almost all known populations of *D. kiusiana* in Korea and a significant part of Japan, which is sufficient to address the proposed issues. We collected 237 leaf samples from five populations in Korea and four populations in Japan. We selected individuals at least 5 m apart to avoid collecting material from relatives, and collected one leaf sample per individual to minimize damage. The collected leaf samples were dried in silica gel and stored at −70 °C in the laboratory of Biological Education, Chonnam National University (BEC), until use. Total genomic DNA was extracted from dried leaf samples using the DNeasy Plant Mini Kit (Qiagen, Seoul, Korea) following the manufacturer’s instructions. The concentration of extracted DNA was determined using a Nano-300 microspectrophotometer (Allsheng, Hangzhou, China) and diluted to 15 ng µL^−1^ to obtain the same concentration of template DNA in each sample for analysis.

### Microsatellite genotyping

We used 16 nuclear microsatellite markers (DKi020, DKi021, DKi022, DKi023, DKi062, DKi070, DKi072, DKi073, DKi082, DKi091, DKi101, DKi104, DKi117, DKi128, DKi129 and DKi131) previously developed for *D. kiusiana* (Lee *et al*., 2017). PCR was performed using a Veriti 96-well thermal cycler (Applied Biosystems, Foster City, CA, USA) in a final volume of 5 µL containing 15 ng of extracted DNA, 2.5 µL Multiplex PCR Master Mix (Qiagen, Valencia, CA, USA), 0.01 µm forward primer, 0.2 µm reverse primer and 0.1 µm of the M13 primer (fluorescently labelled). PCR amplification was performed as follows: initial denaturation at 95 °C for 15 min; 35 cycles of denaturation at 95 °C for 30 s, annealing at 56 °C for 1.5 min and extension at 72 °C for 1 min; and final extension at 72 °C for 10 min. The PCR products were diluted with ddH_2_O (1:30), and 1 µL was analysed on an ABI 3730XL sequencer with GeneScanTM-500LIZTM Size Standard (Applied Biosystems). Allele sizes and peaks for each sample were determined in triplicate using Peak Scanner 2 software (Applied Biosystems) to minimize genotyping errors.

Before analysing the microsatellite data, we estimated the null allele frequency using INEst (inbreeding/null allele estimation) software, which calculates the null allele frequency regardless of the effect of inbreeding (Chybicki and Burczyk, 2009) by running a Markov chain Monte Carlo (MCMC) analysis (number of cycles = 200 000; every *n*^th^ update = 200; burn-in = 2000). This showed that the null allele frequency of all the loci examined ranged from 0.004 % to 0.015 %. Therefore, we used 16 microsatellite markers for statistical analysis.

To ensure that the number of loci was sufficient to analyse the genotype of each individual in the samples, a genotype accumulation curve was applied using the ‘poppr’ (v.2.0.2) package in R (Kamvar *et al*., 2014). This curve shows the ability to discriminate between unique genotypes using an increasing number of molecular markers (Kamvar *et al*., 2015; Capador *et al*., 2018; Inoue *et al*., 2021). The genotype accumulation curve showed that the full recognition (100 %) of all the studied multilocus genotypes could be achieved using 15 loci, and confirmed that 16 loci were sufficient for the identification of individuals (Supplementary Data Fig. S1). We then determined the number of different multilocus genotypes (MLGs) and sorted the homozygous MLGs (HMLGs) that were homozygous at all loci by referring to the raw data.

### Genetic diversity and selfing rate estimation

Genetic diversity parameters were evaluated for each locus based on simple sequence repeat (SSR) allele frequency data using GenAlEx 6.5 (Peakall and Smouse, 2006). These included the number of alleles (*N*_A_), number of private alleles (*P*_A_), observed heterozygosity (*H*_O_), expected heterozygosity (*H*_E_) and inbreeding coefficient (*F*_IS_). Allele richness (*A*_*R*_) and genetic differentiation among populations (*F*_ST_) were determined by calculating the overall fixation index according to the method of Weir and Cockerham (1984) using FSTAT 1.2 (Goudet, 1995). The significance of genotypic population differentiation at each locus and over loci was tested using the log-likelihood-based (G)-based exact test (Goudet *et al*., 1996) in FSTAT. The values of the genetic diversity parameters were then averaged across the population within each group (hereafter ‘lineage’; see details in the Results). Additionally, we calculated genetic diversity parameters at the pooled lineage level.

To estimate selfing rates at the pooled lineage level, we employed ‘R_MES_’ software (robust multilocus estimation of selfing rates). This technique is relatively insensitive to null alleles and scoring errors, which may otherwise lead to the overestimation of selfing rates (see David *et al*., 2007). In addition, empirical studies have confirmed that this method is reliable for estimating selfing rates (Bürkli *et al*., 2017). We estimated the selfing rates using two methods implemented in R_MES_. First, the g_2_ method uses two-locus heterozygosity disequilibrium values and, second, the maximum-likelihood (ML) method maximizes the log-likelihood of the multilocus heterozygosity structure of a sample. As a significance test for selfing rate associated with the g_2_ method, R_MES_ computes the probability that there is no selfing (g_2_ = *s* = 0) obtained from 10 000 iterations of a random assortment of single-locus heterozygosities among individuals. A 95 % confidence interval (CI) for selfing rate provided by R_MES_ was used to determine the significance of selfing rate associated with the ML method.

### Enrichment and sequencing for multilocus cpDNA

Detailed methods are provided in Supplementary Data Method S1.

We targeted 27 non-coding regions of cpDNA using MPM-seq for all samples. This method can provide noteworthy information faster and more cost-effectively in phylogenetic analyses based on independent sources of genomic data (Suyama *et al*., 2021). Notably, we made several modifications to this method as described in Supplementary Data Method S1 with Figs S2 and S3. Finally, we sorted 16 high-productivity regions and used these sequences for analysis (Table S1). The raw reads and haplotype sequences were deposited in GenBank under BioProject ID PRJNA794292 and accession numbers SRX13610178–SRX13610414.

### Genetic structure analysis and phylogenetic inference

For microsatellite analysis, we used a Bayesian clustering approach implemented in STRUCTURE 2.3 (Pritchard *et al*., 2000) using 1 000 000 MCMC iterations (100 000 burn-in with admixture). The simulation used 20 iterations with *K* = 1 to *K* = 9 clusters. The optimal number of clusters, *K*, was determined via the *K* method, using ‘STRUCTURE HARVESTER’ (Earl and vonHoldt, 2012). CLUMPP v.1.1.2 (Jakobsson and Rosenberg, 2007) with a greedy algorithm was used to combine the membership coefficient matrices (Q-matrices) from 1000 iterations for *K *= 2 using random input orders. We also conducted a principal coordinate analysis (PCoA) to determine the genetic structure using the covariance-standardized approach of pairwise Nei’s genetic distances in GenAlEx 6.5. To identify genetic boundaries between the populations, we performed a barrier analysis (Manni *et al*., 2004) based on Monmonier’s algorithm (1973) with 1000 bootstrap matrices of pairwise genetic distances (Nei and Tajima, 1983) that were calculated using ‘MICROSATELLITE ANALYZER’ (MSA) v.4.05 (Dieringer and Schlötterer, 2003). A neighbour-joining (N-J) tree for the populations was constructed using PHYLIP 3.68 (Felsenstein, 2004) based on Nei’s chord distance (*D*_A_) and the proportion of shared alleles (*D*_ps_). Pairwise genetic distances (*D*_A_ and *D*_ps_) were generated with MSA software using a bootstrap analysis of 1000 replicates.

For cpDNA analysis, a parsimony haplotype network was constructed using TCS v.1.21 (Clement *et al*., 2000) with a 99 % connection limit. All indels and inversions were treated as single-point mutations. To investigate the phylogenetic relationships between *D. kiusiana*, including var. *atrocaulis* (MT627481), an ML phylogenetic tree was constructed using Geneious R11.0.5 (Biomatters Ltd, Auckland, New Zealand). We considered two closely related species, *Daphne depauperata* (MW245833) and *D. acutiloba* (MW246150), as the outgroups (Kim *et al*., 2021). Additional data were downloaded from the National Center for Biotechnology Information (NCBI; https://www.ncbi.nlm.nih.gov/). Alignments were performed using ‘MAFFT’ (Katoh and Toh, 2010). The ML analysis was performed using RAxML v.8.2 (Stamatakis, 2014) using default parameters and 1000 bootstrap replicates. For the RAxML tree, the general time-reversible (GTR) model of nucleotide substitution was used along with the gamma model of rate heterogeneity.

### Inference of population demography

To clarify the population size change and divergence histories of the two lineages detected by the structure analysis, an ABC approach was used. ABC enabled us to compare population demographic models and estimate their parameters (Beaumont, 2019). First, we constructed three population size change models, namely a standard neutral model (SNM), population growth model (PGM) and size reduction model (SRM; Supplementary Data Fig. S4a). Then, using the information obtained from the population size change analyses (see details in the Results), three population divergence models (DM1–3) were constructed (Fig. S4b). As the two lineages did not share their ancestries estimated by the structure analysis and cpDNA haplotypes, we did not consider migration between lineages in order to simplify the hypotheses. We used 16 SSR loci datasets, which were converted from fragment size to repeat number prior to the analyses, and calculated the following summary statistics: the averages and standard deviations of the number of alleles, expected heterozygosity, and the allele size range for population size change analyses of both lineages. For the population divergence analysis between the two lineages, in addition to these summary statistics, we calculated the averages of the overall number of alleles, expected heterozygosity, allele size range and *F*_ST_, meaning a total of 16 summary statistics were obtained. The summary statistics were calculated using Arlsumstat v.3.5.2 (Excoffier and Lischer, 2010).

Each model was simulated 10^4^ times with ‘fastsimcoal2’ v.2.6.0.3 (Excoffier and Foll, 2011), and summary statistics were calculated using Arlsumstat. Inbreeding was considered by including the observed fixation index value in the input file of fastsimcoal2. A generalized stepwise mutation model (GSM) was used for the SSR mutation model (Estoup *et al*., 2002). The average mutation rate among loci was set to 5 × 10^−4^ considering the range of mutation rates of SSR, i.e. from 5 × 10^−5^ to 5 × 10^−3^ (Estoup and Angers, 1998; Estoup *et al*., 2002; Marriage *et al*., 2009). GSM was implemented as described by Setsuko *et al*. (2020). All prior distributions of the parameters were generated using R v.4.1.2 (R Core Team, 2021) and their ranges are summarized in Supplementary Data Table S2. Models were compared using the ABC-random forest (RF) approach with 1000 decision trees implemented in the ‘abcrf’ v.1.8.1 package in R (Pudlo *et al*., 2016).

Using the best model selected by ABC-RF, 2 × 10^5^ simulations were repeated, and the nearest 1000 summary statistic values to the observed values were used for the parameter estimation. We used a neural net regression implemented in the ‘abc’ v.2.1 package in R (Blum and François, 2010; Csilléry *et al*., 2012). The logit transformation option was used to maintain the estimated parameter values within their ranges. Posterior mode and 95 % highest posterior density (HPD) were estimated using the ‘density’ function in R and the ‘coda’ v.0.19.4 package in R (Plummer *et al*., 2006), respectively. To convert the timescale from generations ago to years ago, 10 years per generation was used considering the longevity of the target species. By plotting the mode and 95 % HPD over time, the trajectory of population size change for each lineage was obtained (Lu *et al*., 2020). Using 1000 randomly drawn posteriors, a predictive simulation was carried out, and predictive values were compared with the observed values to confirm the goodness-of-fit of the model to the observed data (Gelman *et al*., 2014).

### Ecological niche modelling

We developed a model of the current and historical potential distributions of *D. kiusiana* using Maxent 3.4.4 (Merow *et al*., 2013). Occurrence data were obtained from the Global Biodiversity Information Facility (GBIF; https://www.gbif.org/), including *D*. *kiusiana* var. *atrocaulis* (Rehder) F. Maek., and the sample localities in our investigation. A total of 452 occurrence points were spatially filtered with 25 km between each point using SDMtoolbox 2.4 (Brown *et al*., 2017) to decrease bias when modelling the potential distributions. We used 19 bioclimatic variables (Online Resource 2) obtained for two periods [the present and Last Glacial Maximum (LGM)] from the climatologies at high resolution for the Earth’s land surface areas (CHELSA) dataset (http://chelsa-climate.org; Karger *et al*., 2017). The bioclimatic variables of the LGM are based on three general circulation models (GCMs) – the Community Climate System Model (CCSM4; Gent *et al*., 2011), the Earth System Model based on the Model for Interdisciplinary Research on Climate (MIROC-ESM; Watanabe *et al*., 2011) and the Max Planck Institute for Meteorology Earth System model (MPI-ESM-P). The bioclimate data for 20–45°N and 100–145°E (30 arc-second resolution) were extracted using QGIS 3.16.6. To avoid multicollinearity, we excluded one of the bioclimate variables sharing a high Spearman correlation coefficient (>0.8) using SDMtoolbox 2.4. Therefore, seven of the 19 variables were selected and used to develop the model. To reduce the uncertainty in previous climate models, the LGM distributions from the three GCMs were averaged. Prior to the Maxent runs in batch mode, response curves, jackknife tests, 20 replicates, cloglog output, random seeds, 10 000 background points and 2000 iterations were used. After developing the distribution model, this was projected onto the other two periods of the LGM to estimate the past distribution, and we averaged the LGM distributions of the three GCMs.

### Morphological measurements and statistical analysis

To evaluate the effectiveness of the mechanism to control variability in levels of outcrossing, we explored the floral morphological traits of *D. kiusiana*. A total of 117 samples from eight populations (80 in the west and 37 in the east) were collected during the peak flowering season from 2016 to 2022 (mainly during February and March 2022). During the field survey, the number of flowers was counted by randomly selecting one inflorescence on each individual. Then, we cut the branched stems, including the inflorescences, and brought them to the laboratory at Chonnam National University for subsequent measurements. For each sample, we imaged the longitudinal sections of the flowers using a digital camera (MFX1600; Nahwoo, South Korea) connected to a stereomicroscope (Olympus SZ61; Olympus, Japan). The length and width of the calyx lobe, length of the calyx tube, length of the pistil, and minimum distance between the stigma and anthers were measured using digital calipers built into iworks 2.0 software (Nahwoo) (Supplementary Data Fig. S5). Samples were prepared as voucher specimens by press drying or preserving in 70 % ethanol, and then stored in the National Forest Seed Variety Center of Korea Forest Service (Voucher Nos. NFSV-22-Dk001–110) and the Korea Institute of Oriental Medicine (Voucher Nos. KIOM-19-Dk001–007). Measurements for seven of the samples from the Kyushu population were obtained from dried specimens using the same method as described. All the related images are provided in Supplementary Data Morphological dataset S1.

We compared differences in floral traits related to visibility and herkogamy between the two lineages (west and east). The significance of the differences between the two lineages was confirmed by a statistical *F* test in one-way ANOVA. We also examined the correlations between flower traits and herkogamy in the pooled samples. The degree of herkogamy was estimated as the distance between the shortest stigma and the anther, as their stamens generally consist of two pairs of four. The area of the calyx lobe was roughly estimated by half the length × width. All statistical analyses and significance tests were performed using R v.4.1.2 (R Core Team, 2021).

## RESULTS

### Genetic diversity and selfing rate

Significant differences in genetic diversity were observed between the western and eastern populations (Table 1).

**Table 1.** Genetic variation based on 16 microsatellite loci in nine populations of *Daphne kiusiana.*

Pop. ID	Location	*N*	*G*_type_	*HOMO*_MLG_ (no.)	*R*_Homo_	*N*_A_	*A*_R_	*P*_A_	*H*_O_ (SE)	*H*_E_ (SE)	*F*_IS_
**LI**	**West**										
k-SBI	Sinan-gun, Jeollanam-do	21	21	–	–	41	2.563	7	0.235 (0.059)	0.356 (0.057)	0.314^*^
k-JSI	Sinpyeong-ri, Jeju	25	25	–	–	48	2.920	3	0.358 (0.046)	0.398 (0.038)	0.146^*^
k-JCH	Cheongsu-ri, Jeju	30	30	–	–	42	2.471	3	0.360 (0.043)	0.387 (0.038)	0.062
k-JSE	Seonheul-ri Jeju	28	28	–	–	35	2.120	0	0.283 (0.045)	0.312 (0.046)	0.093
Population mean		26.0	26.0	–	–	41.5	2.518	3.3	0.309 (0.025)	0.363 (0.022)	0.147
Pooled lineage		104	104	–	–	65	4.063	43	0.314 (0.036)	0.484 (0.031)	0.374
**LII**	**East**										
k-GWA	Geoje-si, Gyeongsangnam-do	22	1	*H*_1_ (22)	1.000	16	1.000	1	0.000 (0.000)	0.000 (0.000)	NA
j-SKA	Karatsu-shi, Saga Pref.	22	17	*H*_2_ (3)	0.136	26	1.617	3	0.071 (0.034)	0.153 (0.050)	0.384^*^
j-KYA	Yatsushiro-shi, Kumamoto Pref.	30	8	*H*_3_ (15), *H*_4_ (7), *H*_5_ (2), *H*_6_ (2)	0.867	22	1.363	4	0.000 (0.000)	0.067 (0.033)	1.000^*^
j-MNO	Nobeoka-shi, Miyazaki Pref.	33	17	*H*_7_ (12), *H*_8_ (3), *H*_9_ (3), *H*_10_ (2)	0.606	26	1.570	2	0.021 (0.008)	0.137 (0.047)	0.745^*^
j-MKO	Kobayashi-shi, Miyazaki Pref.	26	16	*H*_11_ (4), *H*_12_ (3), *H*_13_ (3), *H*_14_ (2), *H*_15_(2), *H*_16_(2)	0.615	25	1.560	4	0.012 (0.006)	0.120 (0.054)	0.901^*^
Population mean		26.6	11.8		0.645	23.0	1.422	2.8	0.021 (0.007)	0.095 (0.019)	0.691
Pooled lineage		133	59		0.654	50	3.074	28	0.019 (0.007)	0.301 (0.081)	0.539

*N*, number of individuals; *G*_type_, number of genotypes; *HOMO*_MLG_, homozygous multilocus genotypes; *R*_Homo_, ratio of homozygous individuals at all loci within a population; *N*_A_, number of alleles; *A*_R_, allelic richness; *P*_A_, number of private alleles; *H*_O_, observed heterozygosity; *H*_E_, expected heterozygosity; SE, standard error; *F*_IS_, inbreeding coefficient; NA, not available.

^*^Significant deviation of *F*_IS_ from zero (*P* < 0.01).

**Fig. 2.**
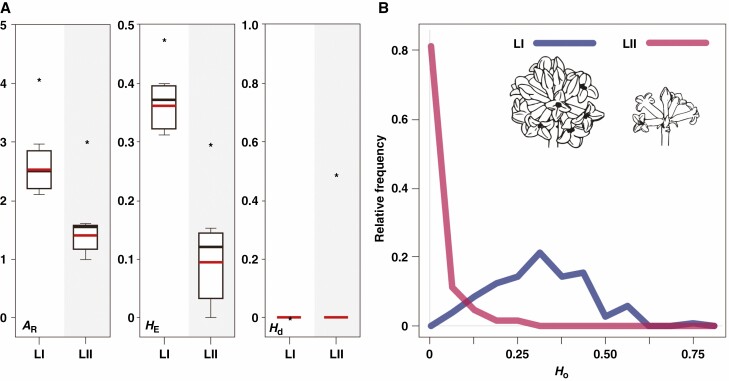
Comparison of genetic diversity between the two lineages of *Daphne kiusiana*. (A) Boxplots showing mean allelic richness (*A*R), expected heterozygosity (*H*E) and haplotype diversity (*H*d). The mean within-population values for each lineage are shown as red lines. Each value of the pooled lineages is marked with an asterisk. (B) Histogram showing the observed heterozygosity (*H*_O_) at the level of individuals for each lineage. LI, lineage I (west); LII, lineage II (east).

Genetic diversity was lower in the eastern lineage (*A*_R_ = 1.422, *H*_E_ = 0.095) than in the western lineage (*A*_R_ = 2.518, *H*_E_ = 0.363). Similarly, the genetic diversity of the pooled eastern lineage (*A*_R_ = 3.074, *H*_E_ = 0.301) was lower than that of the pooled western lineage (*A*_R_ = 4.063, *H*_E_ = 0.484) (Fig. 2A).

Based on the genotyping of 237 individuals, 87 were homozygous at all loci and they consisted of 16 MLGs (homozygous multilocus genotypes, hereafter ‘HMLGs’). HMLGs occurred only in the eastern lineage, where one or a few genotypes dominated each population (0.136–1.000), and the observed heterozygosity was extremely low (Fig. 2B). In addition, none of the HMLGs in the eastern lineage were shared across populations. By contrast, in the western lineage, all individuals had different genotypes, and HMLGs did not appear. *F*_ST_ values evaluated between the populations within each lineage were higher in the east (mean = 0.712) than in the west (mean = 0.291), and were significant over all loci (*P* < 0.001) (Table 2).

**Table 2.** Summary statistics of selfing rate and genetic differentiation. The selfing rate was estimated from empirical datasets using the *g*_2_ and maximum likelihood (ML) methods. For the *g*_2_ method, estimated selfing rate and *P*-values for the condition *H*_o_:*s* = 0 are given. For the ML method, the hypothesis *H*_o_:*s* = 0 was tested by model simplification, comparing deltadev to a chi-square with one degree of freedom.

Lineage	*g*_2_ method		ML method			*F*_ST_
	Selfing rate	*P*-value	Selfing rate	δdev_s = 0_	95 % CI	
I	0.178	**0.002**	0.129	**3.92**	[0.0015–0.2455]	0.291^*^
II	0.920	**0.000**	0.914	**32.07**	[0.8559–0.9448]	0.712^*^

Values in bold type indicate that the selfing rate is significantly different from 0. The selfing rate was estimated from each pooled lineage. *F*_ST_ values are the means of genetic differentiation among populations for each lineage.

^*^Significant differentiation where *P* < 0.001.

Notable differences in selfing ratios were observed between the western and eastern populations. Selfing rates by the g_2_ method and ML method at the lineage level were considerably higher in the east, averaging 92.0 and 91.4 % compared to 17.8 and 12.9 % in the west, respectively (Table 2).

### Phylogeographical structure

**Fig. 3.**
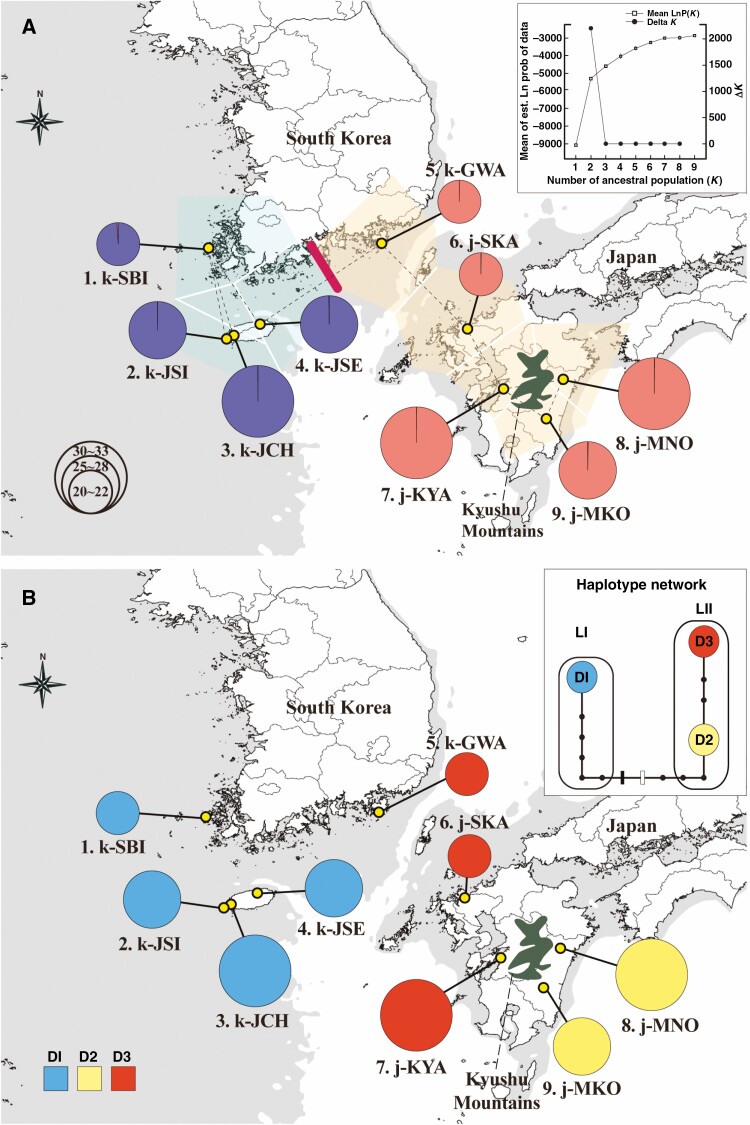
Genetic composition of *Daphne kiusiana* populations. (A) STRUCTURE clustering using 16 microsatellite loci (*K* = 2) and barrier analysis based on Monmonier’s algorithm and significance, tested using means of 1000 bootstrap matrixes of *D*_A_ genetic distance (Nei *et al*., 1983). The rate of change in the log-likelihood probability and Δ*K* based on the estimated number of genetic clusters (*K*) is shown in the upper-right inset. (B) Geographical distribution of three chloroplast haplotypes based on 16 non-coding cpDNA regions. The grey shading represents exposed coastal areas and sea basins during times of glacially induced alterations in sea level during the Late Pleistocene.

**Fig. 4.**
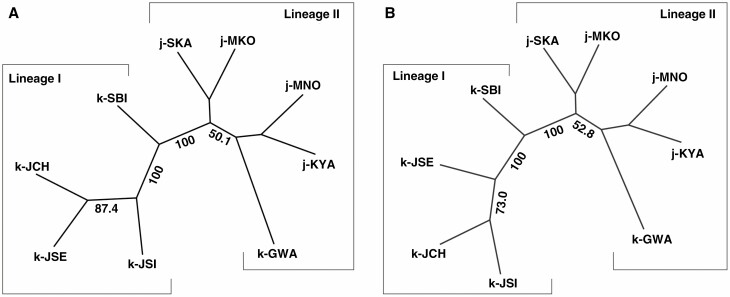
Phylogenetic relationships (neighbour-joining trees) for nine populations of *Daphne kiusiana* based on (A) Nei’s chord distance (*D*_A_) and (B) the proportion of shared alleles (*D*_ps_). Values in the tree branches are percentage bootstrap values estimated from 1000 reiterations. Bootstrap support at internodes are shown if values are >50 %.

In the microsatellite analysis, the STRUCTURE, Barrier, PCoA and N-J trees used to infer the population structure of *D. kiusiana* consistently revealed that the two lineages are genetically distinct, i.e. the western lineage (LI) and the eastern lineage (LII). In the STRUCTURE analysis, while the mean likelihood scores increased progressively with *K* values of 1–9, ∆*K* values computed for all *K* classes indicated a strong signal at *K* = 2 (Fig. 3A). At *K* = 2, which was the optimal number of clusters, a strong genetic structure was found among the populations, divided clearly into the two lineages (Fig. 3A). We also made bar plots of individual ancestry from *K* = 3 to *K* = 9 (Supplementary Data Fig. S6). Barrier analyses based on pairwise *F*_ST_ identified a strong barrier, which was clearly divided into the west and east lineages (Fig. 3A). The results of the PCoA for individuals also revealed this separation (Fig. S7). The N-J trees based on the *D*_A_ and *D*_ps_ genetic distances classified the nine populations into two lineages with a remarkably high bootstrap value of 100 (Fig. 4).

For the multilocus cpDNA analysis, the cpDNA sequence was aligned with a consensus length of 8904 bp, and polymorphisms were identified in seven non-coding regions (*ndh*F-*rpl*32, *mat*K-*trn*K, *rps*11-*rps*8, *trn*G-*atp*A, *trn*E-*trn*T, *ycf*4-*cem*A and *atp*B-*rbc*L). Fourteen polymorphisms (11 nucleotide substitutions and three indels) were identified, and three haplotypes (D1–D3) were determined. Each population harboured only a single haplotype. Consistent with the results of the microsatellite analysis, the haplotype network showed a linear topology, indicating that *D. kiusiana* was clustered into two different lineages (Fig. 3B). LI comprises haplotype D1, and LII harbours haplotypes D2 and D3. The ML tree revealed that haplotype D1 and var. *atrocaulis* were monophyletic, with a 65 % bootstrap value, whereas D2 and D3 were completely monophyletic. This indicates that, in the phylogeographical history, LI and LII were independent lineages.

### Past population size change and population divergence

The average classification errors of the ABC-RF model selection ranged from 0.354 to 0.388 (Table 3). Although these error rate values were not low in all analyses, as votes by RF were concentrated into a single model and the posterior probabilities were relatively high (0.702–0.802), we considered that our ABC-RF model selection was reliable. Based on the goodness-of-fit of the best model, some predicted standard deviation parameters deviated from the observed parameters, although most of the summary statistics were well predicted (Supplementary Data Figs S8 and S9). Therefore, we conclude that our parameter estimation also performed well.

**Table 3.** Model comparison using an approximate Bayesian computation random forest (ABC-RF) approach.

Analysis	Lineage	Proportion of votes by RF^a^						Posterior probability	Error rate
		SNM	PGM	SRM	DM1	DM2	DM3		
Population size change	I	0.023	0.264	**0.713**	–	–	–	0.802	0.354
Population size change	II	0.080	0.208	**0.712**	–	–	–	0.702	0.354
Population divergence	–	–	–	–	0.086	0.157	**0.757**	0.731	0.388

^a^The best model is shown in bold type.

SNM, standard neutral model; PGM, population growth model; SRM, size reduction model; DM, divergence model.

**Fig. 5.**
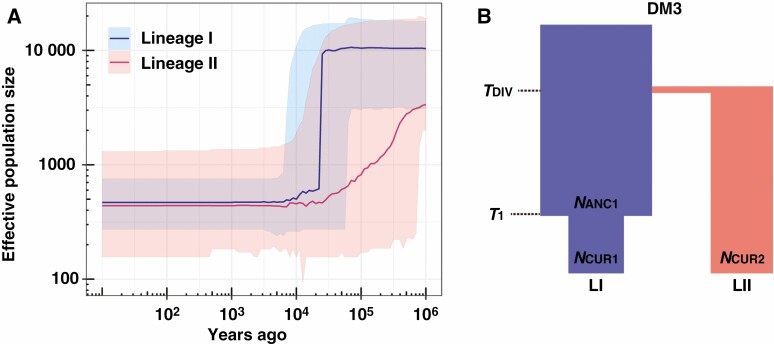
Inferred demographic history for *Daphne kiusiana*. (A) Trajectories of effective population size change in lineage I and lineage II predicted by the size reduction model. Lines and coloured areas indicate the posterior mode and 95 % highest posterior density. (B) The best supported population divergence model in ABC analysis of the genetic data sets. *N*_CUR_, current effective population size; *N*_ANC_, ancestral effective population size; *T*, event time for population size change; *T*_DIV_, divergence time.

In the population size change analyses, the SRM was selected as the best model for both lineages (Table 3). Based on the posterior modes of *T* (in thousand years ago, kya), a probable bottleneck occurred within LI at 12.1 [4.9–67.1] kya, while within LII at 54.1 [10.4–999.3] kya (Table 4). A severe bottleneck effect is clearly more evident in predominantly outcrossing LI than in predominantly selfing LII (Fig. 5A). The trajectories of population size change showed that although both lineages reduced in size in the Late Pleistocene, the reduction in LI was more rapid and recent than that in LII (Fig. 5A). During the Holocene, both lineages showed similar and very low effective sizes, with posterior mode values of 468 and 439 for LI and LII, respectively (Table 4). In the population divergence analysis, divergence model 3 (DM3) was selected as the best model (Table 3 and Fig. 5B) and the posterior mode (95 % HPD) of the divergence time between the two lineages (*T*_DIV_) was estimated to be 21.3 (12.8–70.4) kya (Table 4).

**Table 4.** Posterior mode and 95 % highest posterior density (HPD) of parameters of the best model.

Analysis	Population size change		Population divergence
	Lineage I	Lineage II	
Best model	SRM	SRM	DM3
*N*_CUR_	468 (272–753)	439 (156–1311)	–
*N*_ANC_	10 354 (3137–18 018)	3364 (2001–19 054)	–
*T*_1_ (kya)	12.1 (4.9–67.1)	54.1 (10.4–999.3)	–
*T*_DIV_ (kya)	–	–	21.3 (12.8–70.4)
*shape*	1.74 (0.84–4.30)	0.52 (0.50–0.93)	1.03 (0.53–2.37)
*P*_GSM_	0.15 (0.00–0.36)	0.31 (0.07–0.46)	0.28 (0.06–0.50)

SRM, size reduction model; DM3, divergence model 3.

*N*_CUR_, current effective population size; *N*_ANC_, ancestral effective population size; *T*_1_, event time for population size change; *T*_DIV_, divergence time; *shape* and *P*_GSM_, generalized stepwise mutation model parameters.

### Ecological niche modelling

**Fig. 6.**
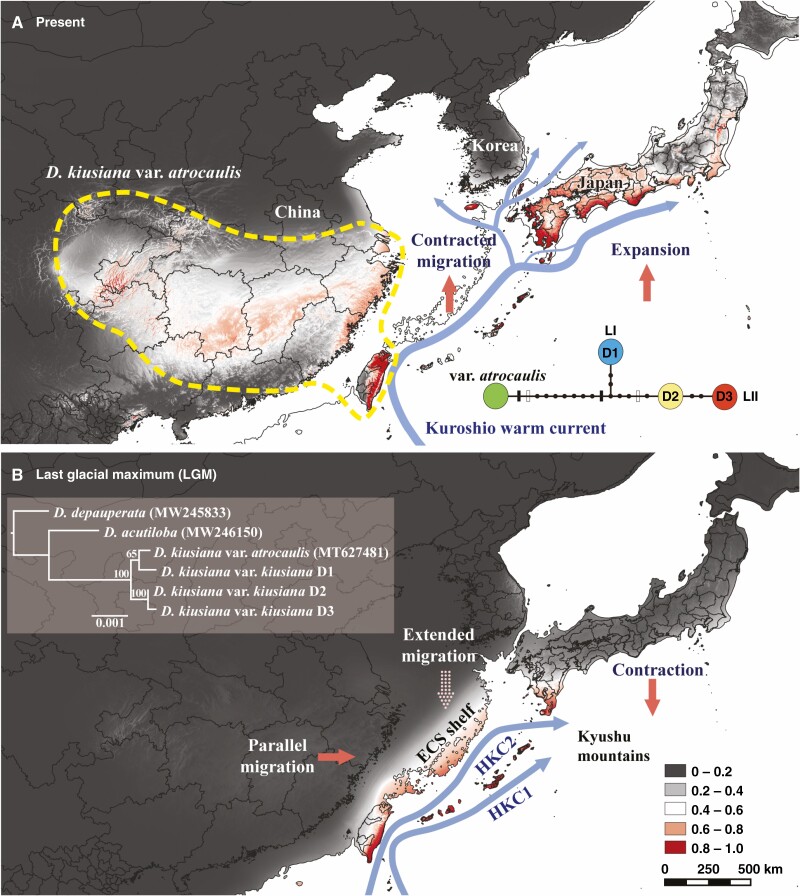
Potential distributions of *Daphne kiusiana* predicted by ecological niche modelling. The potential range during the LGM was averaged from three general circulation models. (A) Present; chloroplast haplotype network based on 16 non-coding cpDNA regions of var. *kiusiana* and var. *atrocaulis*. (B) LGM; ML tree is boxed in grey. HKC 1 indicates the main track of the Kuroshio Warm Current (proposed by Ujiie *et al*., 2003; Kao *et al*., 2006; Zheng *et al*., 2016). The scale bar in the ML tree represents the average number of nucleotide substitutions per site. HKC 2 is the hypothetical Kuroshio Warm Current (proposed by Vogt-Vincent and Mitarai, 2020).

The current distribution model of *D*. *kiusiana* (Fig. 6A) showed a high average AUC (area under the ROC curve; 0.906). The highest contributing variable was ‘bio14’ (precipitation of driest month, 57 % contribution) followed by ‘bio04’ (temperature seasonality, 12.0 %) and ‘bio02’ (mean diurnal range, 11.9 %). The current distribution model was significantly consistent with warm-temperate climate zones extending from western Japan to Guizhou in China, including the southern edge of the Korean Peninsula and northern Taiwan. The potential distribution during the LGM was revealed with a high probability (>0.6) along the palaeo-coastline from Taiwan to the southern East China Sea (ECS) shelf, southern Kyushu and the Ryukyu Islands of Japan (Fig. 6B).

### Differences in floral traits: visibility and herkogamy

**Fig. 7.**
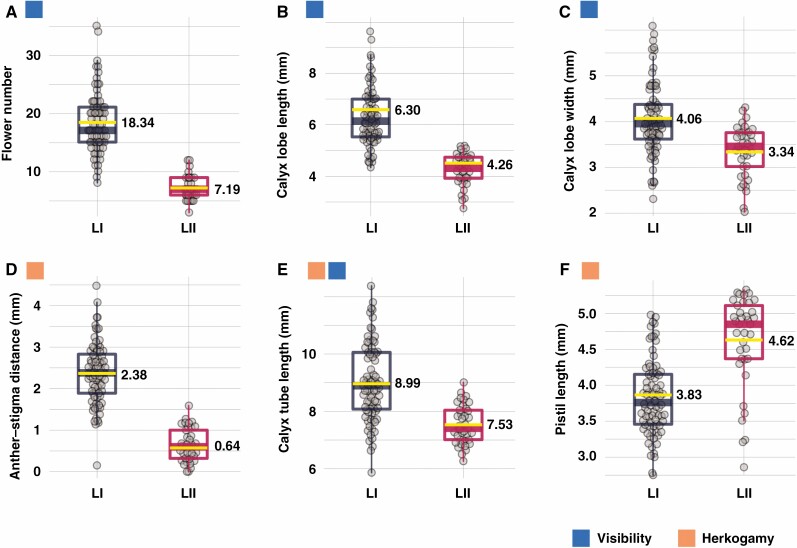
Boxplots expressing morphological variations in two *Daphne kiusiana* lineages (LI and LII). The box signifies the distribution of the 25–75 % quartiles, and the median is represented by a horizontal line within the box. The ends of the vertical lines indicate minimum and maximum data values, respectively. Mean values are represented by a yellow line, with the value shown next to the line. The differences between the two lineages were significant for each respective trait where *P* < 0.001 (*F* test).

**Fig. 8.**
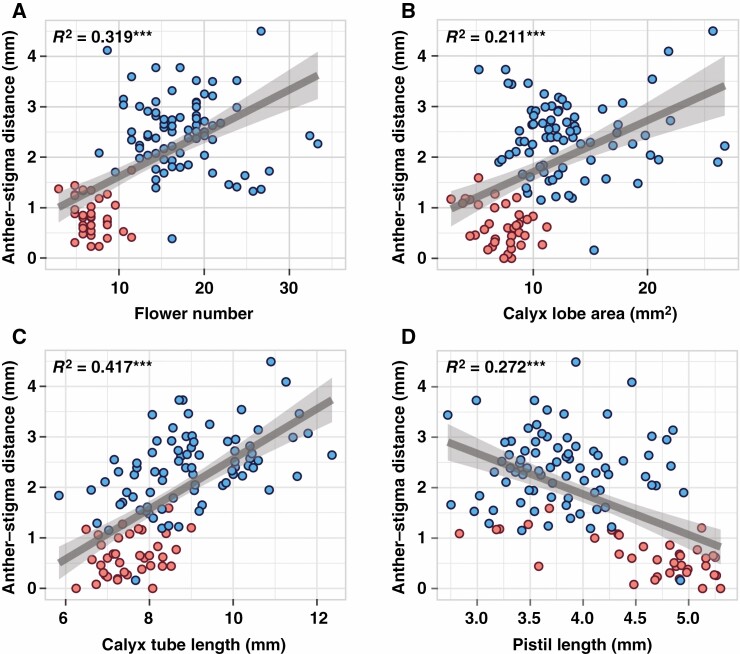
Scatter plots showing correlations between herkogamy and floral morphological traits of *Daphne kiusiana*. Blue and red circles represent the data of lineage I (LI) and lineage II (LII), respectively. Grey straight line, fitted values; shaded area, 95 % confidence interval; ***, *P* < 0.001.

We found significant differences in floral visibility and herkogamy between the two lineages. The floral visibility of LI was significantly lower than that of LII. This pattern was evidenced by flower number (*F*_1,115_ = 160.3, *P* < 0.001; Fig. 7A), calyx lobe length (*F*_1,115 _= 107.6, *P* < 0.001; Fig. 7B), calyx lobe width (*F*_1,115_ = 25.1, *P* < 0.001; Fig. 7C) and calyx tube length (*F*_1,115_ = 39.03, *P* < 0.001; Fig. 7E), which affected the increase in the surface area of the inflorescence. A significant difference in anther–stigma distance was also observed between the two lineages (*F*_1,115_ = 180.5, *P* < 0.001; Fig. 7D). Significant differences were also found in the lengths of the calyx tube and pistil (*F*_1,115_ = 50.38, *P* < 0.001; Fig. 7F), the floral organs that make up the herkogamy trait (Fig. 7). Significant positive correlations were found between anther–stigma distance and flower number (*R*^2^ = 0.319, *P* < 0.001; Fig. 8A), calyx lobe area (*R*^2^ = 0.211, *P* < 0.001; Fig. 8B) and calyx tube length (*R*^2^ = 0.417, *P* < 0.001; Fig. 8C). However, a negative relationship between anther–stigma distance and pistil length was observed (*R*^2^ = 0.272, *P* < 0.001; Fig. 8D), indicating that a reduction in the distance between the stigma and anthers occurred via an increase in the length of the pistils.

## DISCUSSION

To our knowledge, this is the first study to characterize in detail the evolutionary history and demographic dynamics associated with the selfing syndrome in a wild shrub with subcapitate inflorescences. The eastern lineage (LII) of *D. kiusiana*, which has smaller and fewer flowers, was characterized by a predominantly unique occurrence of HMLGs, lower genetic diversity and higher genetic differentiation compared to those of the western outcrossing lineage (LI). Our results suggest that the selfing lineage in *D. kiusiana* has been largely driven by gradual directional selection towards lower levels of floral visibility and herkogamy for efficient self-pollination in response to historical environmental changes. The transition to selfing accompanied by morphological modifications may have been triggered by severe competitive interactions among/within species. Intrinsic factors of *D. kiusiana*, such as its life-history traits and niche in the ecological succession, may also have been important in facilitating its evolution. Thus, the selfing syndrome lineage or species may exhibit a demographic signature of a gradually reduced effective population size, as evidence of adaptation.

### Demographic genetic consequences: selfing vs. outcrossing

We found that the eastern populations (LII) had significantly higher homozygosity than the western populations (LI). This pattern is typical of the genetic characteristic observed in selfing species/populations (Duncan and Rausher, 2013; Voillemot and Pannell, 2017) and is consistent with theoretical predictions (Busch and Delph, 2012; Wright *et al*., 2013). Indeed, R_MES_ allowed the estimation of selfing rates in our study species and revealed that the relative ratio of LII was significantly higher. We also found that HMLGs at all loci occurred only in LII. One or a few HMLGs dominate each population and coexist with several rare genotypes. This is consistent with the genetic patterns in selfing populations in which a few HMLGs dominate specific habitats (Shibayama and Kadono, 2008; Triest *et al*., 2021). Therefore, we suggest that the genetic characteristics of the LII populations are the result of selfing. This suggestion is strongly supported by our results that show genetically less diversity and higher differences among the LII populations, which are typical indications of selfing populations (Charlesworth and Pannell, 2001; Duminil *et al*., 2009; Voillemot and Pannell, 2017).

On the other hand, although it is highly unlikely, as many flowering plants flexibly combine sexual and asexual reproduction systems, our results related to HMLGs may open the possibility for asexual reproduction, such as vegetative propagation and apomixis. However, these plants are inherently heterozygous and consistently maintain their original genetic diversities. Moreover, heterozygosity is expected to increase under clonal propagation owing to the accumulation of mutations over long-term evolutionary progress (Birky, 1996; Balloux *et al*., 2003; and also see the Japanese populations discussed by Zhu *et al*., 2020). To the best of our knowledge, there have been no reports of asexual reproduction in this species, and its impact on genetic consequences is therefore considered limited.

### Evolutionary history of selfing lineage

Our STRUCTURE, Barrier analysis, PCoA, N-J tree, cpDNA ML tree and ENM analyses all point to the fact that LI and LII have independently evolved. In addition, considering previous studies on other evergreen trees belonging to similar forest biomes (Cao *et al*., 2018; Han *et al*., 2020), we have reasonably inferred the geographical location of their ancestral populations during the LGM. These populations would have been distributed in separate regions along the palaeo-coastline, including (1) the ECS shelf (LI) and (2) the southern tip of Kyushu (LII). Consequently, the two extant lineages may have resulted from high-latitude expansion from different source populations in response to dynamic changes in land configuration and the contemporary flow of the Kuroshio Warm Current (Fig. 6). Therefore, to gain insight into what boosted the selfing rates sufficiently enough to cause the floral changes, we inferred historical and ecological events likely to have occurred during the last glacial period.

It is worth noting that progressively exposed ECS shelves could have provided new habitat for LI during the last glacial period. Historical circumstances would have been favourable for the ancestors of western populations during the last glacial period. An extensive shelf, such as the ECS basin exposed to climate cooling, would have provided new open habitats for LI. The migration process appears to have progressed along with the development of ecological succession (probably primary succession); plants would have progressively colonized the ECS shelf and predominated the shrub layers of the forests. Indeed, the abundance of congeneric evergreen shrubs often increases in open areas (Sakata and Nakahama, 2018). Climate cooling would not be beneficial but the relative evolutionary selection pressures for the transition to selfing would have been small because of the reward of colonizing the ECS shelf. Subsequently, a severe population bottleneck of LI arose during the high-latitude migrations in the Holocene with rapid sea-level increases.

In contrast, the Japanese islands would not have allowed the populations to expand further south because there was relatively little Pacific coastline retreat (Lee *et al*., 2013*b*; Cao *et al*., 2018). This may imply conflicting selection between the two lineages with respect to their mating systems. For the eastern populations, the climate cooling would have restricted them to the southern tip of Kyushu, and probably forced them to compete with numerous cold-tolerant species, presumably migrated downwards from the Kyushu Mountains (i.e. competitive interaction; Cheptou and Dieckmann, 2002). Furthermore, mating competition in early flowering *D. kiusiana* may have been accelerated by limited pollinators (Barrett, 1992). Under these conditions (i.e. rare mates and/or rare pollinators), selfing can be favoured because it offers reproductive assurance (Darwin, 1876; Pannell and Barrett, 1998; Busch and Delph, 2012) and boosts seed production (Davis and Delph, 2005). The selfing syndrome may evolve under ecological conditions that demanded relatively strong competitive interactions among and within species. Intrinsic factors of *D. kiusiana* may also have played an important role in facilitating this evolution. Notably, the life-history traits of this shrub are characterized by early flowering (i.e. lack of resources for pollination; Kalisz and Vogler, 2003), and bird-mediated seed dispersal.

### Demographic dynamics and adaptive significance of selfing syndrome

Similar findings showing that independently evolved selfing species/lineages frequently show lower levels of floral visibility (Sicard and Lenhard, 2011; Goodwillie and Weber, 2018) and herkogamy (Takebayashi *et al*., 2006; Busch and Delph, 2012) compared to their outcrossing ancestors suggest that these alterations can have adaptive significance. Moreover, selfing can promote trait divergence for adaptation (Hodgins and Yeaman, 2019). As a result of morphological analysis for *D. kiusiana*, quantitative values such as flower number, calyx tube length and calyx lobe area decreased in predominant selfing LII. This suggests that many traits associated with the reduced surface area of the inflorescence are responsible for the reduced visibility. Moreover, the pistil length in plants of LII derived from LI was notably increased. This result is remarkable because it implies that the energy used to improve floral visibility was reallocated to increase pistil length, resulting in a low level of herkogamy. In addition, these plants have flowers in which the filaments of the stamens and calyx tube are fused, which can be effective by simultaneously lowering visibility and herkogamy levels (Figs 7E and 8C). Therefore, by combining resource consumption, structural complexity and functional efficiency, the flowers of LII may represent a gradual by-product that required sufficient time to evolve. The evolution of the selfing syndrome may be limited by genetic drift alone, without gradual adaptation.

In *Capsella*, the short timescales of speciation suggest that the extensive phenotypic evolution was driven rapidly by genetic drift over about the last 20 000 years (Foxe *et al*., 2009) or last 50 000 years (Guo *et al*., 2009). However, these inferences raise the question of when the flower changes began in relation to the species divergence time. Similar to previous studies, our estimate of the divergence time of the two *D. kiusiana* lineages was 21 300 years ago, and signs of a bottleneck event were found in both lineages. However, we found that the pattern of effective population size reduction shown in the ABC-derived trend lines was different between the two morphologically contrasting lineages. The selfing eastern lineage (LII) shows demographic stability, whereas the predominantly outcrossing western lineage (LI) is associated with a severe population bottleneck. In detail, the effective population size of LI remained stable for a relatively long period but showed a dramatic decline during the Holocene. In contrast, the effective population size of LII decreased in a gradual manner during the last glacial period and then remained stable from the LGM to the present (Fig. 6). As the rate of selfing increased, many of these populations may have maintained a size that can slightly offset the negative effects of genetic drift. This implies that the transition to selfing accompanied by the morphological modifications can evolutionarily stabilize reduction of the effective population size.

The SRM in LII estimated the event time of the size reduction to be ~54 100 years ago, during the last glacial period (ca. 110–10 kya; Huang and Schaal, 2012; Alvarez-Solas *et al*., 2019). The effective population size reached a minimum during the LGM. These results suggest that the evolution of characteristic selfing flowers in *D. kiusiana* may have taken place from the onset of the last glacial that could have triggered intense competitive interactions. In this context, functional and morphological changes to the flowers for effective self-pollination may have been almost completed at the LGM. These inferences are consistent with the hypothesis that flower size decreased in the *Capsella* selfing lineage before its extensive geographical spread (Sicard *et al*., 2011). It is therefore reasonable that the evolutionary timescale includes the starting point at which the morphological changes begin, slightly further back in time than estimated by previous studies (i.e. Foxe *et al.*, 2009; Guo *et al*., 2009). The last glacial period may have provided ample time for purifying energetically expensive traits that attract or reward pollinators, as well as for reallocating energies for effective self-pollination.

## Supplementary Material

mcac142_suppl_Supplementary_Data_S1

mcac142_suppl_Supplementary_Data_S2

mcac142_suppl_Supplementary_Figures

mcac142_suppl_Supplementary_Tables

## SUPPLEMENTARY DATA

Supplementary data are available online at https://academic.oup.com/aob and consist of the following. Table S1: The developed 16 cpDNA markers of *Daphne kiusiana* used in the analysis. Table S2: Prior distribution of compared population size change and population divergence models. Figure S1: Genotype accumulation curve for 16 microsatellite loci in *Daphne kiusiana*. Figure S2: Schematic diagram of the construction of the multiplexed sequencing library of cpDNA. Figure S3: Schematic diagram of the identification of variations in the cpDNA non-coding regions. Figure S4: Comparison of population size change and population divergence models. Figure S5: All floral measurements made to characterize variations in floral morphology. Figure S6: Bayesian individual-based clustering of *Daphne kiusiana* populations at *K* = 3 to *K* = 9. Figure S7: Principal coordinate analysis based on Nei’s genetic distance. Figure S8: Goodness-of-fit of the size reduction model. Figure S9: Goodness-of-fit of the population divergence model 3. Method S1: Multiplex sequencing for cpDNA: MPM-seq with modification. Dataset S1: Measurement images of floral morphological characters of *Daphne kiusiana*.
