# Supplementary material for: Genetic and demographic signatures accompanying the evolution of the selfing syndrome in Daphne kiusiana, an evergreen shrub
Source: Ann Bot. 2022 Dec 5;131(5):751–67. doi: 10.1093/aob/mcac142 (PMC10184445; doi:10.1093/aob/mcac142)
Supplement: mcac142_suppl_Supplementary_Data_S1 [file mcac142_suppl_supplementary_data_s1.docx]

**Methods S1**: **Multiplex sequencing for chloroplast DNA: MPM-seq with modification.**

***Primer design***

We first designed 27 primer pairs using the cpDNA genomic data of *D. kiusiana* obtained from a sample collected from Jeju Island, Korea (Cho *et al*., 2018; GenBank Accession No. KY991380). In the complete chloroplast genome sequence of *D. kiusiana* (171,491 bp), we excluded the inverted repeat (IR) regions (a pair of 41,891 bp) to avoid multiple amplicons; primers were designed by selecting sites in the non-coding regions (intergenic space and intron) with an expected amplification length of ca. 600–1,000 bp in a large single copy (LSC) region (85,028 bp) and a small single-copy (SSC) region (2,681 bp). We utilized Primer3 v. 0.4.0 (Rozen and Skaletsky, 2000) and the Geneious program in R11.0.5, according to the following parameters: primer size = 18–22 bp, melting temperature (TM) = 53–60 °C, and GC content = 35–65%.

To perform multiplexed sequencing of cpDNA, sequence tails referenced to the MIG-seq primer sequence for the 1st PCR (Suyama and Matsuki, 2015) were added to the designed primer sequences ([Supplementary Data](https://oup.silverchair-cdn.com/oup/backfile/Content_public/Journal/aob/129/6/10.1093_aob_mcac049/1/mcac049_suppl_supplementary_material.docx?Expires=1656911782&Signature=3BecZIfbSHZ-PwuolF~h1stfWynm3LfN0A1epRP~xzAkfsSX~qtzysnlepONviTPNZEtrWpOoSKBacc-CFQoCaSBsnVY-msow0kXulRptKvQd8j3RtkmwageNqDJOpzOc4kps3BMknVnFdwk4iEg6MwrKL7KqVb8mxKHOkB9L3ZCkidQRiiOqTK-Yv84lEZEKX6GFVPqjfPmE4dSyKSSNIYbAp3WRmxwfWj5S3DmPDjLoQdDj-7dDGg5BaFV75W4aBr-v2HMgLiSl9xDc8sJy81cEJK4VQDpKbCzSMb4hM2~3bZXd42-NFc7MsdfoDeM7I4oOFqmDpAepFIQtCbWjw__&Key-Pair-Id=APKAIE5G5CRDK6RD3PGA)Fig. S2). The sequence tails comprised a portion (14 bases) of the Illumina adapter sequence and an anchor for the 2nd PCR primers (3 bases). In total, the tailed CP primers for the 1st PCR consisted 5′ tail (14 bp) + anchor for the 2nd PCR primers (3 bp) + CP primer (18–22 bp). The 27 pairs of tailed CP primers comprised three sets (nine pairs per set), and each set was assigned to evenly cover the range of the expected amplification lengths.

***Library construction***

The multiplexed sequencing library was prepared using the MPM-seq method (Suyama *et al*., 2021) with modification to the 1st and 2nd PCR steps. The 1st PCR step was performed to amplify the non-coding region of cpDNA with tailed CP primers instead of the MIG-seq primer set-1. The volume of the PCR reaction mixture per sample was 7μl, containing 1μl of template DNA, 0.18 μM of each 1st PCR primer (nine pairs), and 3.5 μl of 2 × Multiplex PCR Master Mix (QIAGEN, Seoul, Korea). PCR amplification was performed by initial denaturation at 95 °C for 15 min followed by 20 cycles of denaturation at 95 °C for 30 s, annealing at 56 °C for 1.5 min and extension at 72 °C for 1.5 min, and final extension at 72 °C for 10 min using a GeneAmp® PCR System 2700 Thermal Cycle (Applied Biosystems, Foster City, CA, USA). The 1st PCR products were visualized on 0.8% agarose gels stained with RedSafe (120 V, 25 min).

The 2nd PCR step was performed to add complementary sequences for the binding sites of the Illumina sequencing flow cell and to independently add individual indices to each sample using the indexed forward and reverse primers. The 1st PCR product from each sample was diluted with deionized water (1:20) and used as the template. The 2nd PCR was performed in a 10-μl reaction mixture containing 2 μl of diluted 1st PCR product, 0.250 U of 5 × PrimeSTAR GXL Buffer (Takara Bio, Kusatsu, Japan), 200 μM of dNTP mixture, 0.2 μl of PrimeSTAR GXL DNA Polymerase (Takara Bio), and 0.2 μM of indexed forward and reverse primer. The PCR conditions were as follows: 15 cycles of denaturation at 98 °C for 10 s, annealing at 54 °C for 15 s, and extension at 68 °C for 1 min.

After the two-step PCR process, the libraries from each sample—each with a different pair of indices—were pooled in equal volumes. The mixed libraries were purified using a QIAquick PCR Purification Kit (Qiagen, Seoul, Korea). Fragments in the size range of 700–1,200 bp in the purified library were then isolated using the Pippin Prep DNA size selection system (Sage Science, Beverly, MA, USA).

***Sequencing and Selection of CP primers***

Multiplexed libraries were sequenced on the Illumina MiSeq platform (LAS, Gimpo, Korea) using a MiSeq Reagent kit v3 (300 cycles; Illumina). Both ends of the non-coding region were read using paired-end sequencing (reads 1 and 2). This generated 7,376,630 paired-end reads (301 × 301 bp). Each of the 237 samples was identified using a dual index, and the raw reads of each indexed sample were grouped. The amplified reads for each sample were 24,756–143,696 (mean = 62,250). The sequences of each of the 237 samples of *D. kiusiana* were deposited in the GenBank database (BioProject ID PRJNA794292; BioSample accession numbers SAMN24619490–SAMN24619726).

The reads were analyzed using Geneious 11.0.5 program. The paired reads of each sample were mapped to the reference genome of *D. kiusiana* with medium sensitivity, were suitable for NGS, did not trim, and had a minimum mapping quality of 30. As a result of mapping, 20,697–140,713 reads (mean = 57,566) were aligned for each individual, and the total pairwise identity was 97.64%. To select the non-coding region of cpDNA for analysis, we excluded the regions that were considered PCR error, i.e., where both ends of the non-coding region were insufficiently amplified (< 5 minimum depth of coverage) and where the amplified regions were not appropriate. In addition, to address sequencing error, the quality of all bases constituting the aligned column of the sequenced region was checked by pairwise identity. We then excluded the regions with sequencing errors where the pairwise identity of the amplified region was < 90%. Based on these criteria, we selected 16 non-coding regions with an average of 1,553.17 amplification reads and a pairwise identity of 96.73%. The characteristics of the 16 chloroplast DNA markers are presented in [Supplementary Data](https://oup.silverchair-cdn.com/oup/backfile/Content_public/Journal/aob/129/6/10.1093_aob_mcac049/1/mcac049_suppl_supplementary_material.docx?Expires=1656911782&Signature=3BecZIfbSHZ-PwuolF~h1stfWynm3LfN0A1epRP~xzAkfsSX~qtzysnlepONviTPNZEtrWpOoSKBacc-CFQoCaSBsnVY-msow0kXulRptKvQd8j3RtkmwageNqDJOpzOc4kps3BMknVnFdwk4iEg6MwrKL7KqVb8mxKHOkB9L3ZCkidQRiiOqTK-Yv84lEZEKX6GFVPqjfPmE4dSyKSSNIYbAp3WRmxwfWj5S3DmPDjLoQdDj-7dDGg5BaFV75W4aBr-v2HMgLiSl9xDc8sJy81cEJK4VQDpKbCzSMb4hM2~3bZXd42-NFc7MsdfoDeM7I4oOFqmDpAepFIQtCbWjw__&Key-Pair-Id=APKAIE5G5CRDK6RD3PGA)Table S1.

To confirm the variation in cpDNA, the nucleotide sequences were identified as the consensus sequence based on the aligned sequence in which the reads amplified by each primer were aligned. The consensus sequence was determined by consensus threshold highest quality (with base matching of at least 60%) for each nucleotide. Random errors appearing in only one read of the alignment column were not reflected in the consensus sequence ([Supplementary Data](https://oup.silverchair-cdn.com/oup/backfile/Content_public/Journal/aob/129/6/10.1093_aob_mcac049/1/mcac049_suppl_supplementary_material.docx?Expires=1656911782&Signature=3BecZIfbSHZ-PwuolF~h1stfWynm3LfN0A1epRP~xzAkfsSX~qtzysnlepONviTPNZEtrWpOoSKBacc-CFQoCaSBsnVY-msow0kXulRptKvQd8j3RtkmwageNqDJOpzOc4kps3BMknVnFdwk4iEg6MwrKL7KqVb8mxKHOkB9L3ZCkidQRiiOqTK-Yv84lEZEKX6GFVPqjfPmE4dSyKSSNIYbAp3WRmxwfWj5S3DmPDjLoQdDj-7dDGg5BaFV75W4aBr-v2HMgLiSl9xDc8sJy81cEJK4VQDpKbCzSMb4hM2~3bZXd42-NFc7MsdfoDeM7I4oOFqmDpAepFIQtCbWjw__&Key-Pair-Id=APKAIE5G5CRDK6RD3PGA)Fig. S3). The nucleotide sequences of the selected noncoding regions were concatenated for each sample. cpDNA haplotypes were determined based on the concatenated sequence alignments.

**LITERATURE CITED**

**Cho WB, Han EK, Choi G, Lee JH. 2018.** The complete chloroplast genome of *Daphne kiusiana*, an evergreen broad-leaved shrub on Jeju Island. *Conservation Genetics Resources* **10**: 103–106. doi: 10.1007/s12686-017-0774-5.

**Rozen S, Skaletsky H. 2000.** Primer3 on the WWW for general users and for biologist programmers. In: Misener S, Krawetz A, eds. *Bioinformatics Methods and Protocols.* New Jersey: Humana Press, 365–386.

**Suyama Y, Hirota SK, Matsuo A, et al. 2021.**Complementary combination of multiplex high‐throughput DNA sequencing for molecular phylogeny. Ecological Research **37**: 171–181.  [doi: 10.1111/1440-1703.12270](https://doi.org/10.1111/1440-1703.12270).

**Suyama Y, Matsuki Y. 2015.** MIG-seq: an effective PCR-based method for genome-wide single-nucleotide polymorphism genotyping using the next-generation sequencing platform. *Scientific Reports* **5**: 1–12. doi: 10.1038/srep16963.
