# Supplementary material for: Genetic and demographic signatures accompanying the evolution of the selfing syndrome in Daphne kiusiana, an evergreen shrub
Source: Ann Bot. 2022 Dec 5;131(5):751–67. doi: 10.1093/aob/mcac142 (PMC10184445; doi:10.1093/aob/mcac142)

**Morphological dataset S1: Measurement images of floral morphological characters of *Daphne kiusiana*.**


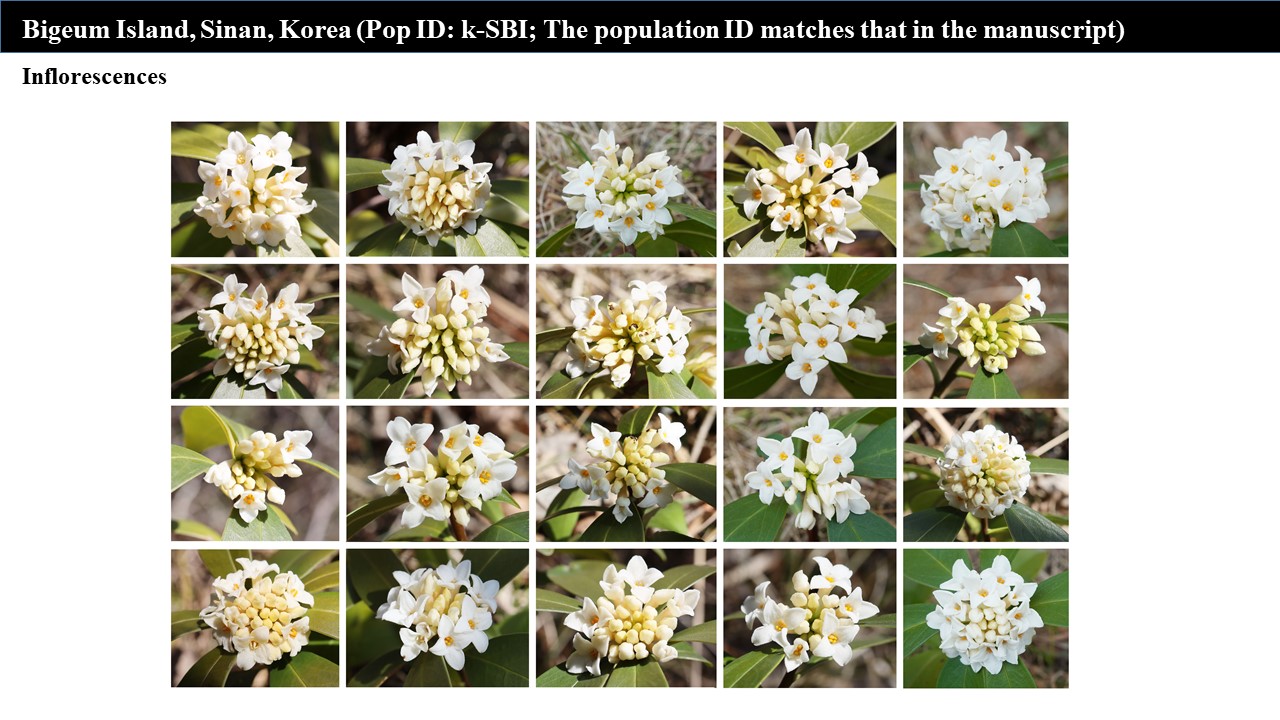


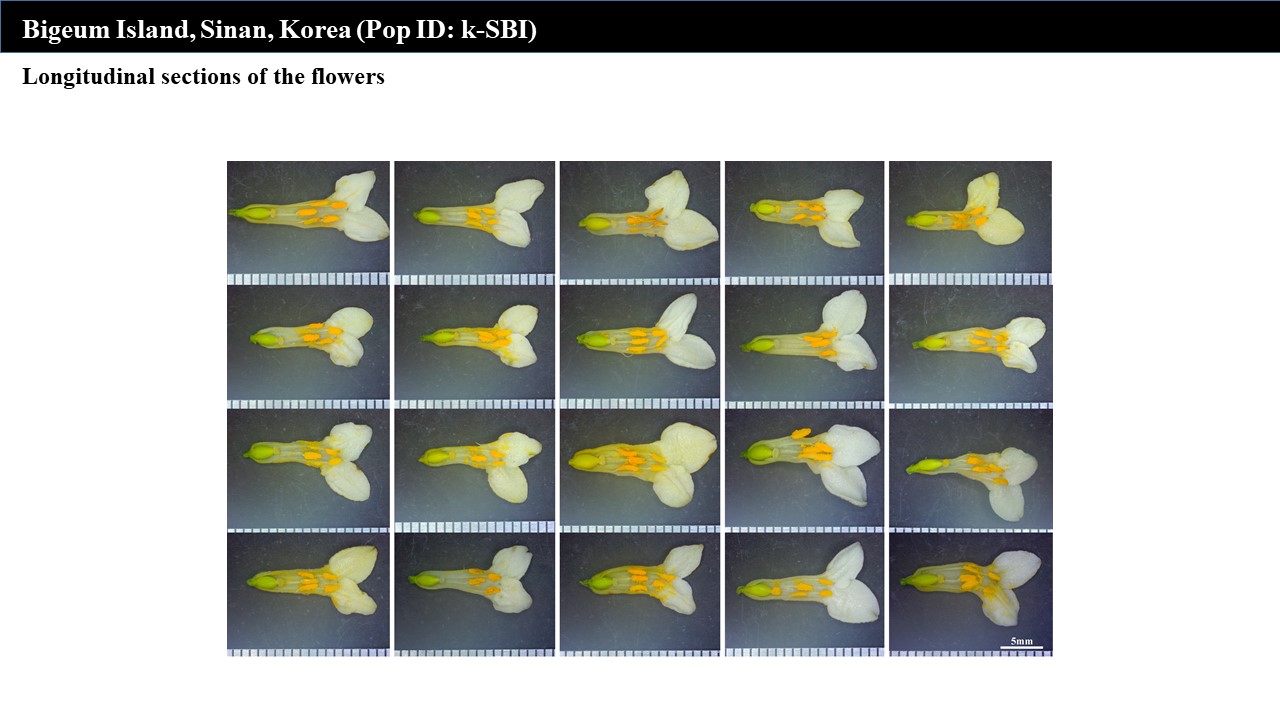


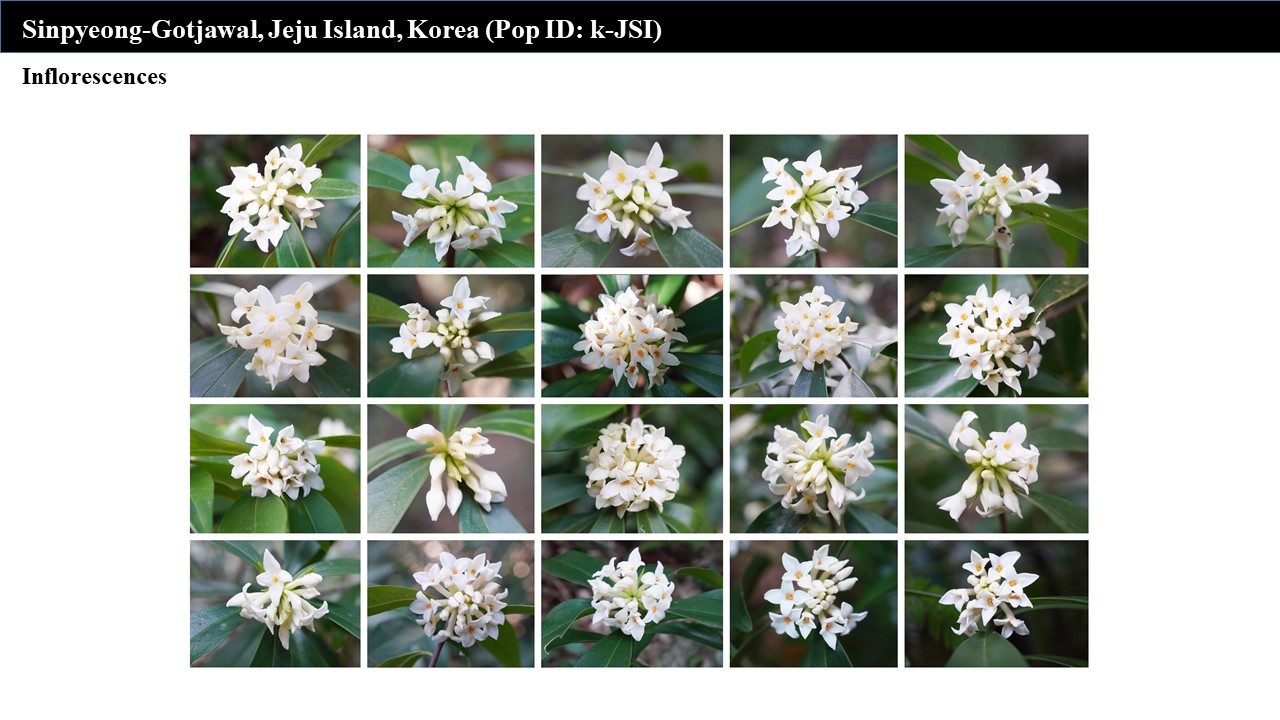


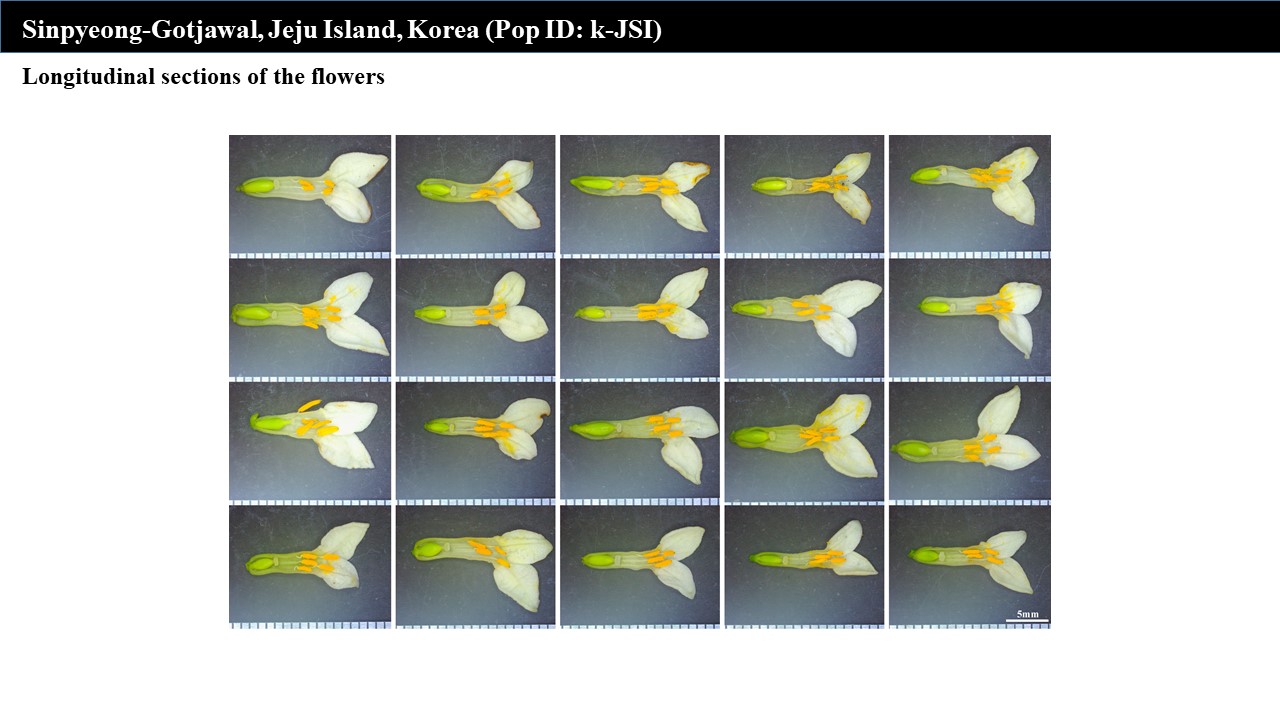


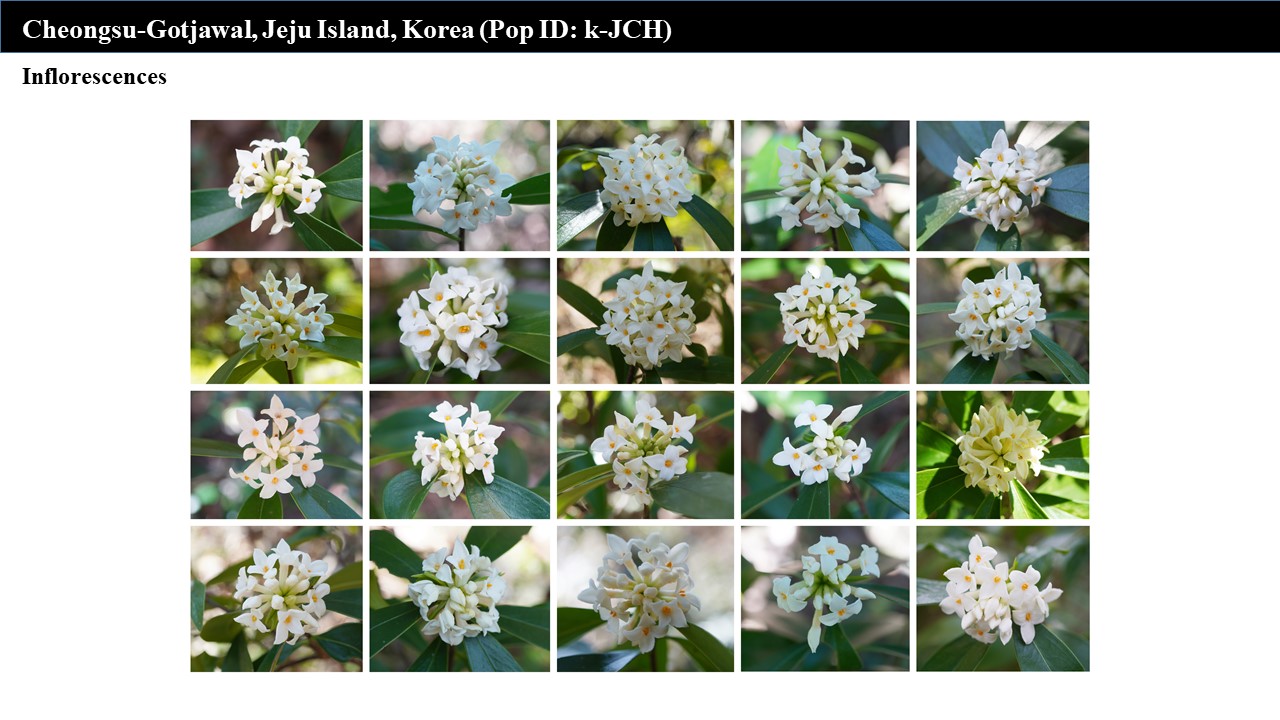


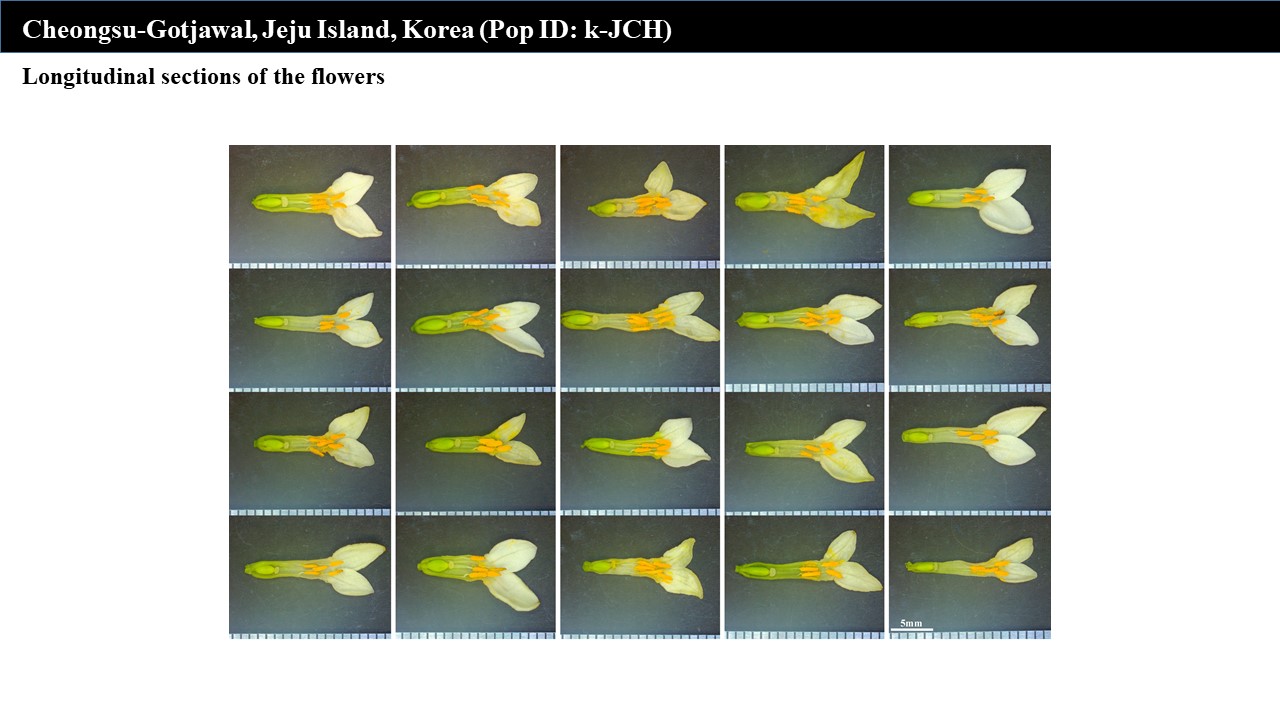


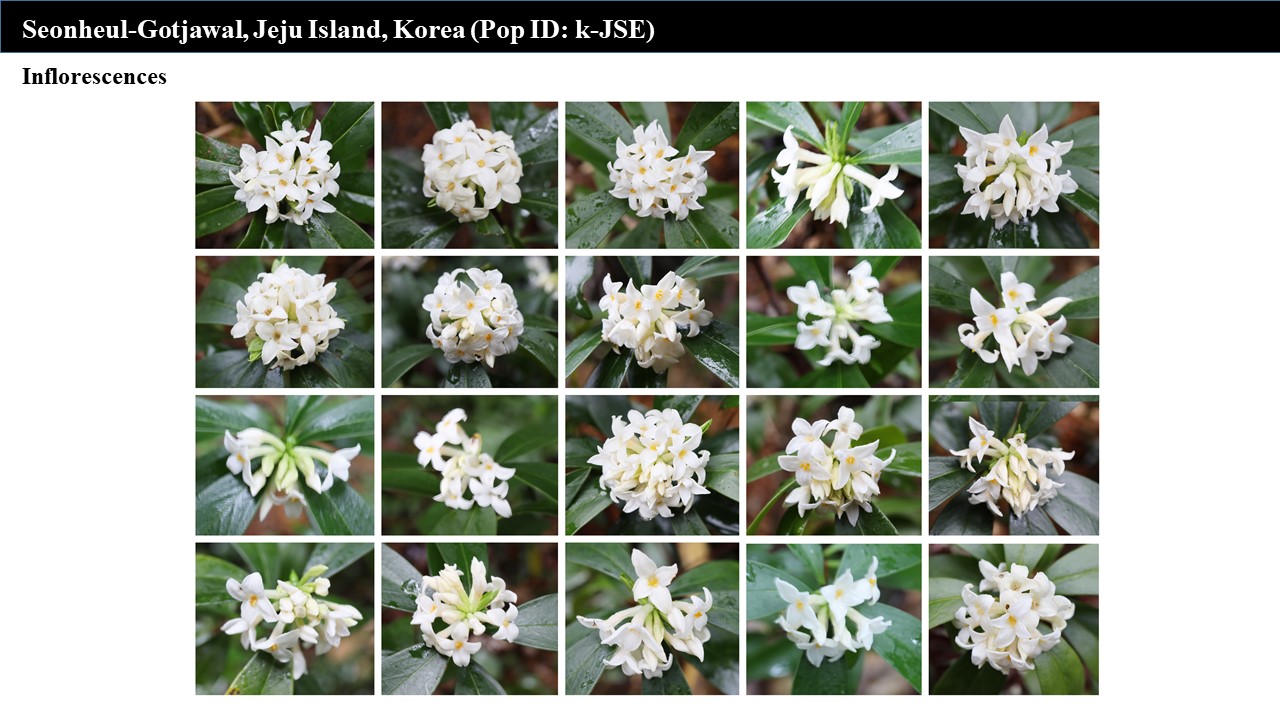


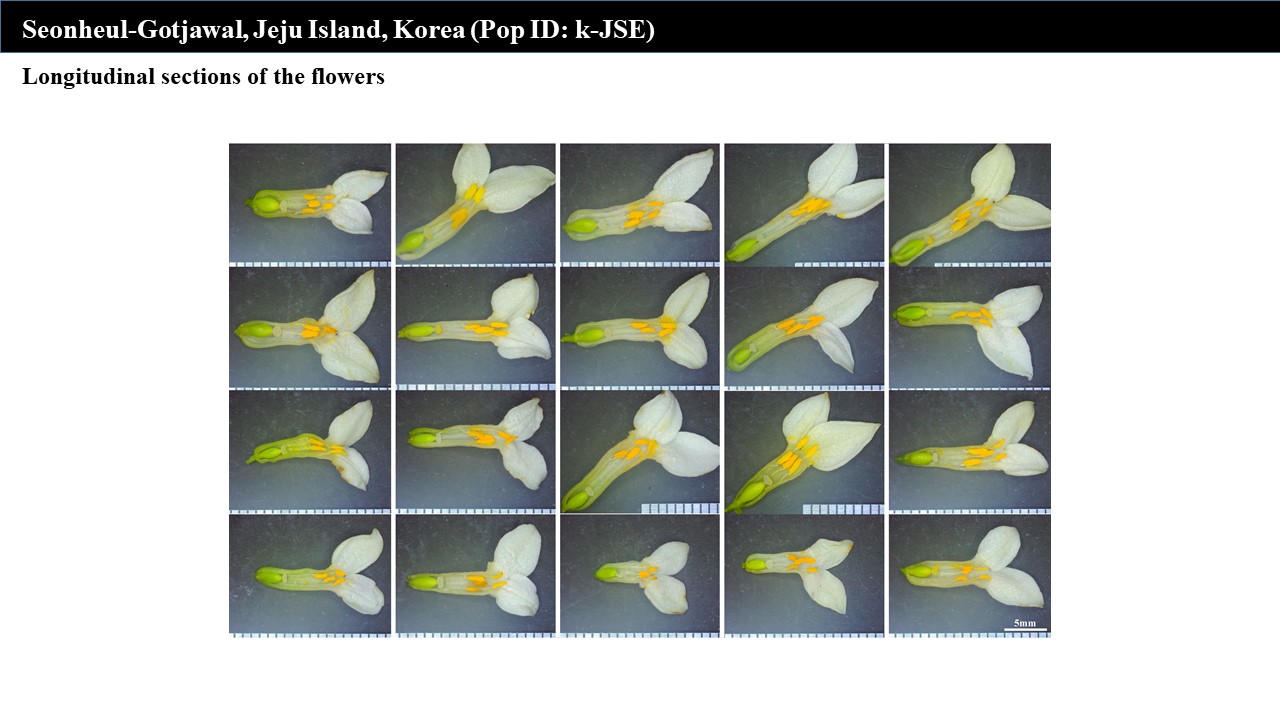


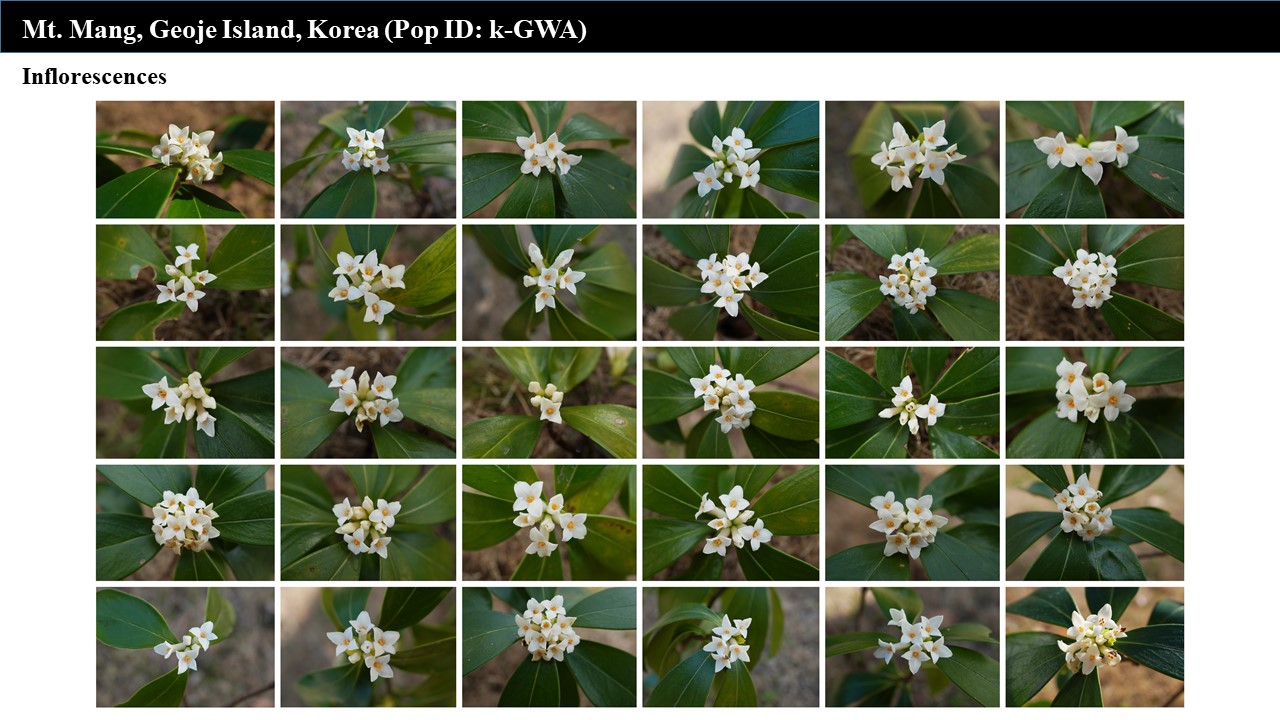


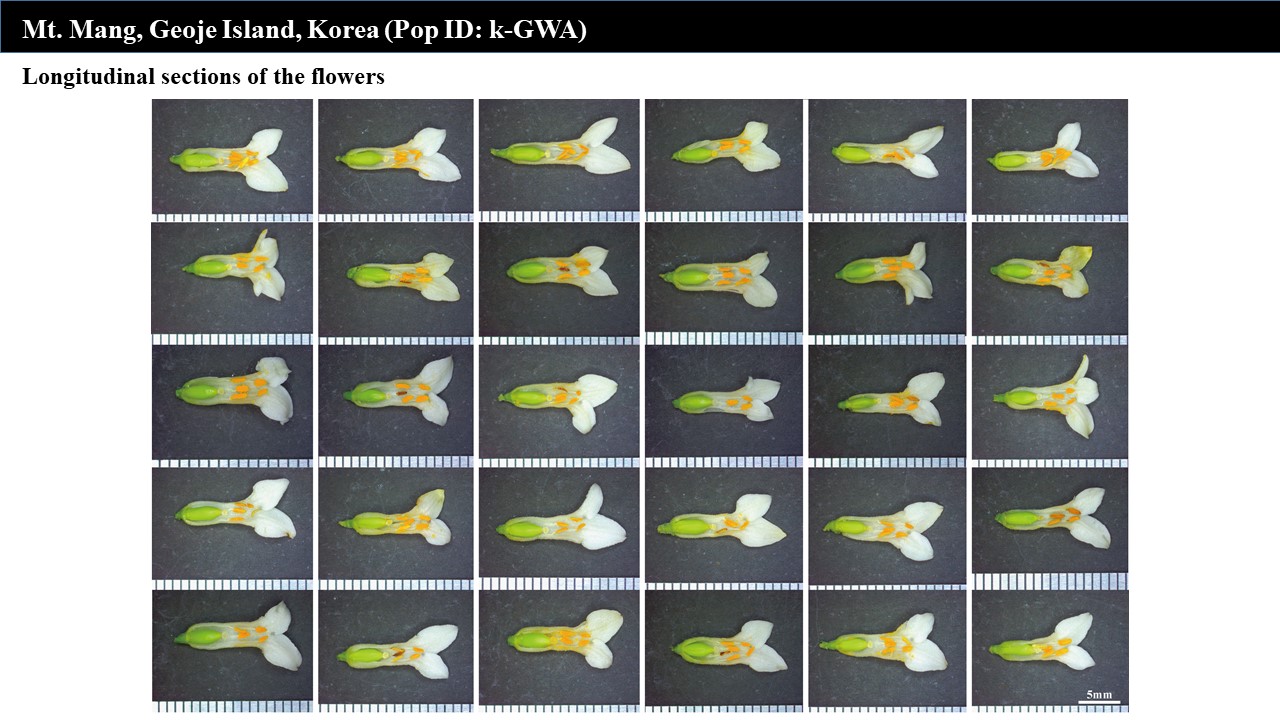


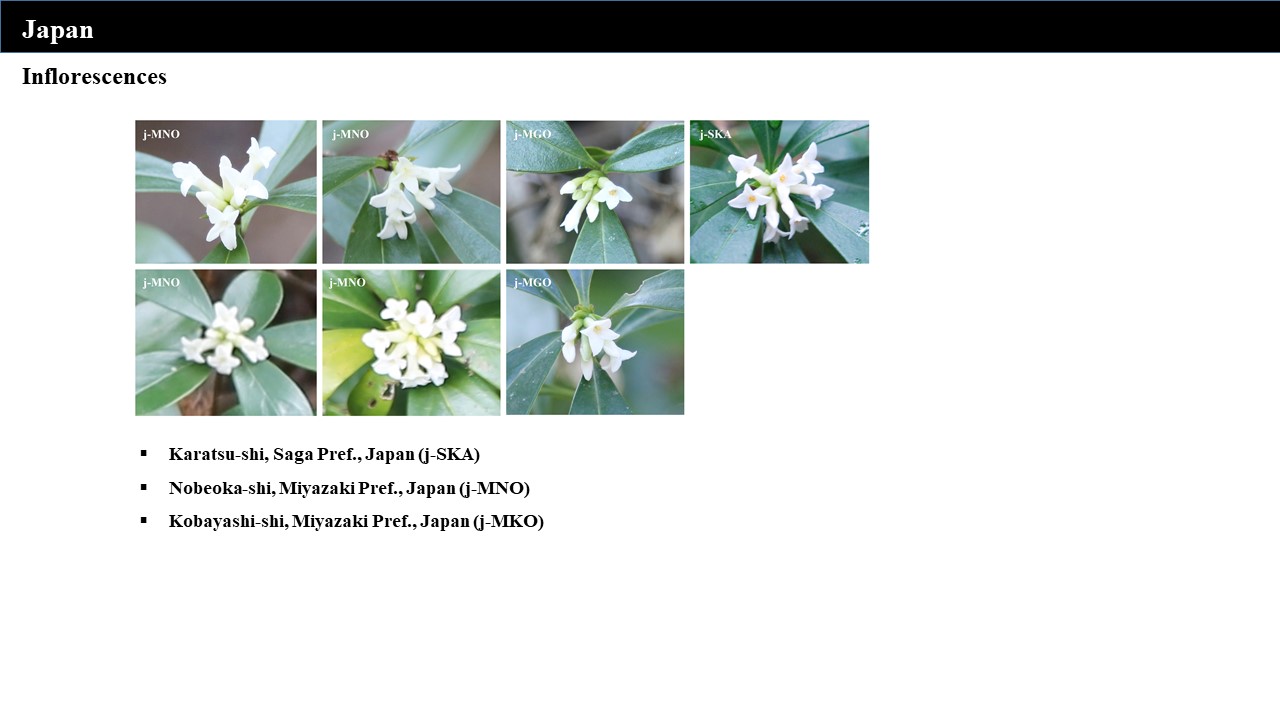


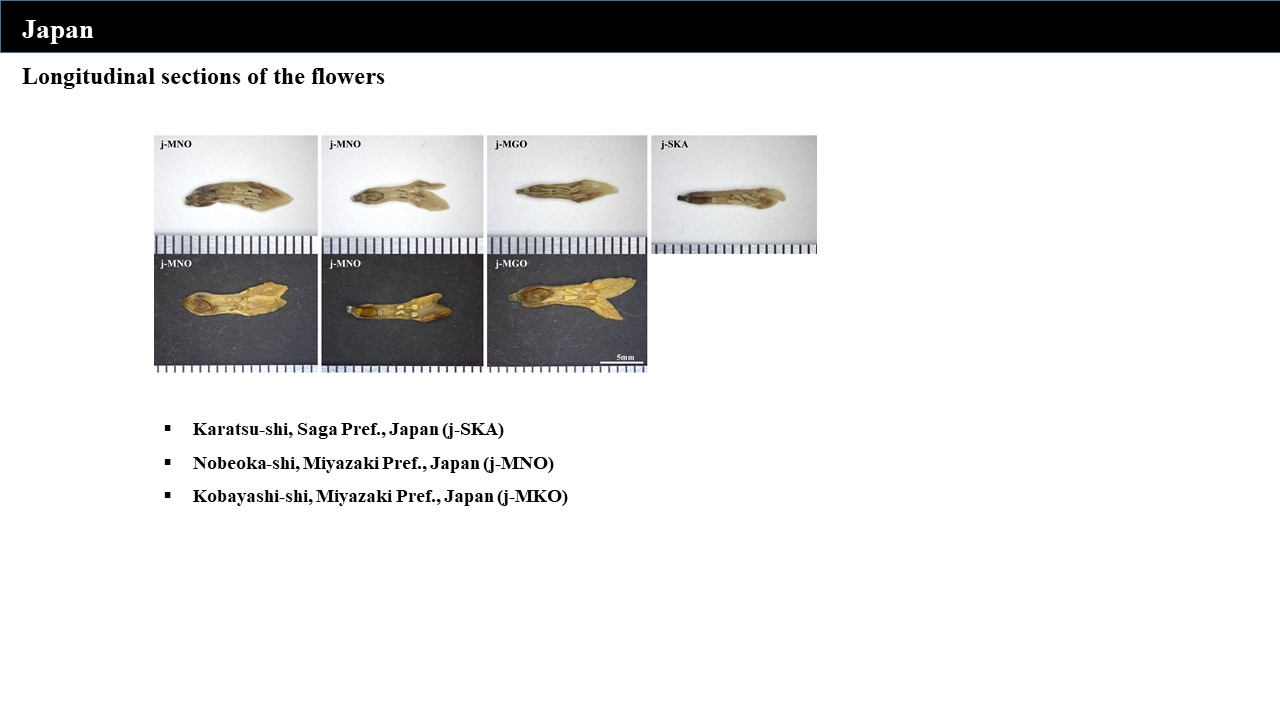

Supplement: mcac142_suppl_Supplementary_Data_S2 [file mcac142_suppl_supplementary_data_s2.docx]
