# Supplementary material for: Genetic and demographic signatures accompanying the evolution of the selfing syndrome in Daphne kiusiana, an evergreen shrub
Source: Ann Bot. 2022 Dec 5;131(5):751–67. doi: 10.1093/aob/mcac142 (PMC10184445; doi:10.1093/aob/mcac142)
Supplement: mcac142_suppl_Supplementary_Figures [file mcac142_suppl_supplementary_figures.docx]

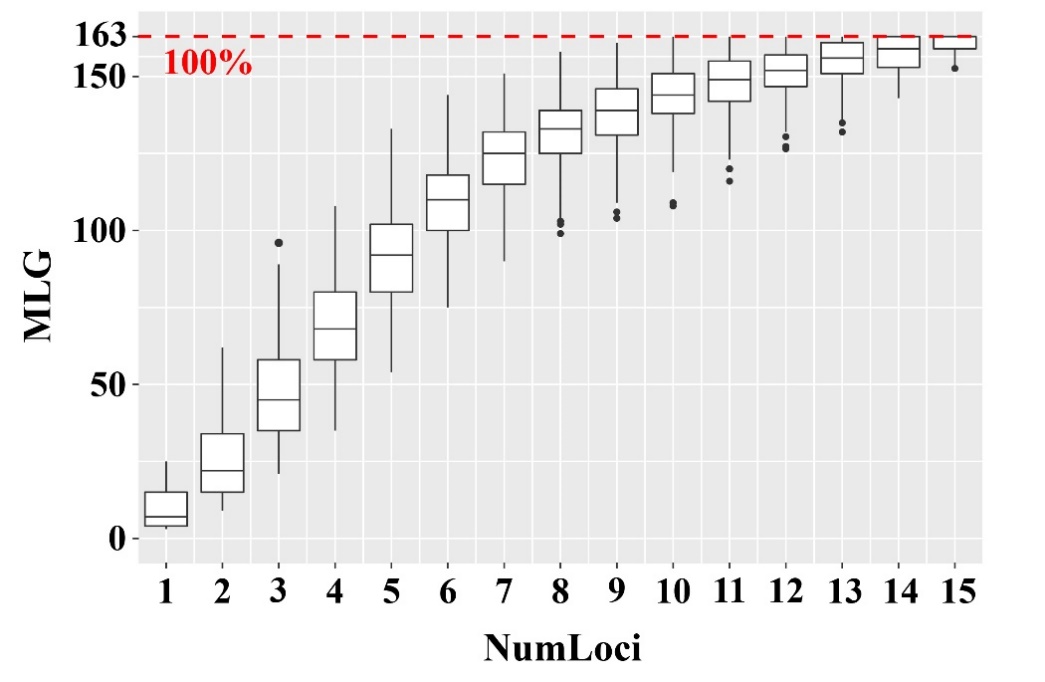


**Figure S1:** **Genotype accumulation curve for 16 microsatellite loci in *Daphne kiusiana*.** Numbers of observed multilocus genotypes (MLGs) are denoted on the vertical axis from 0 to the observed 163 MLGs in the *D. kiusiana* populations. Numbers of loci are denoted on the horizontal axis. Boxplots each contain 1,000 random samples representing different possible combinations of *n* loci. The MLG resolution of 100% is indicated by the horizontal dashed line.

**
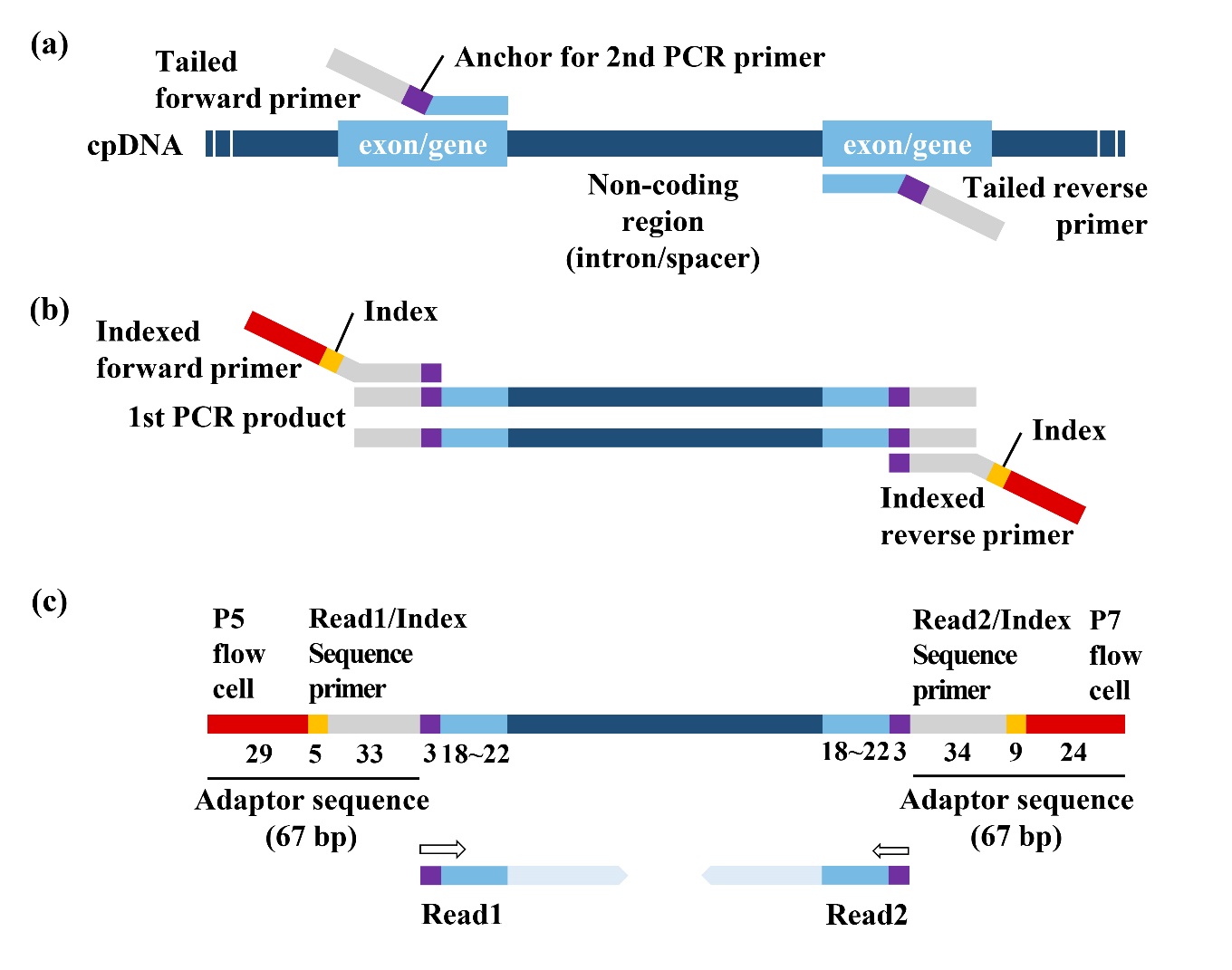
**

**Figure S2: Schematic diagram of the construction of multiplexed sequencing library of cpDNA.** (a) Multiple non-coding regions of cpDNA were amplified by multiplex PCR using developed tailed cpDNA primers (1st PCR). (b) The 1st PCR products were subsequently used as the templates for the 2nd PCR (tail PCR). The 2nd PCR enabled the addition of the index of each sample to the 1st PCR product using indexed forward and reverse primers. (c) Each 2nd PCR product was pooled into a single mixture library. The mixture was then purified, size-selected, and used for Illumina paired-end sequencing (read 1 and 2) and index reading. The sequence of the generated library consisted of binding sites for the P5 flow cell oligonucleotide, binding sites for read 1 and index sequencing primers, forward cpDNA primer, non-coding region, reverse cpDNA primer, binding site of read 2 and the index sequencing primer, and P7 flow cell oligonucleotides.

**
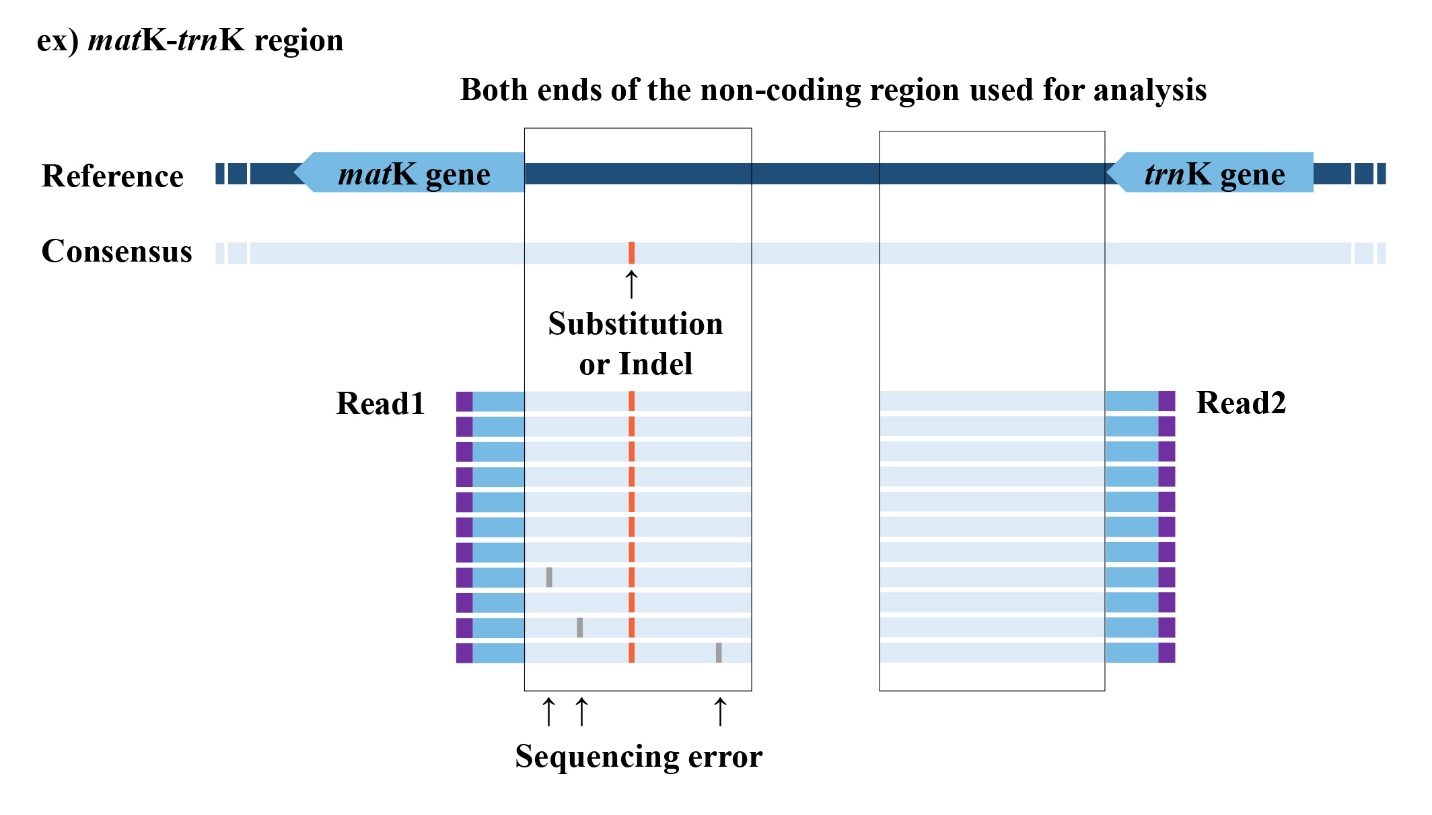
**

**Figure S3: Schematic diagram of the identification of variations in the cpDNA non-coding regions.** cpDNA regions were selected by checking the number of reads mapped to both ends of the non-coding region and the total % pairwise identity of the nucleotide sequence. The variations of cpDNA were detected in the sequence determined by a consensus threshold of 60% for each nucleotide excluding random errors that appeared in only one read.

**
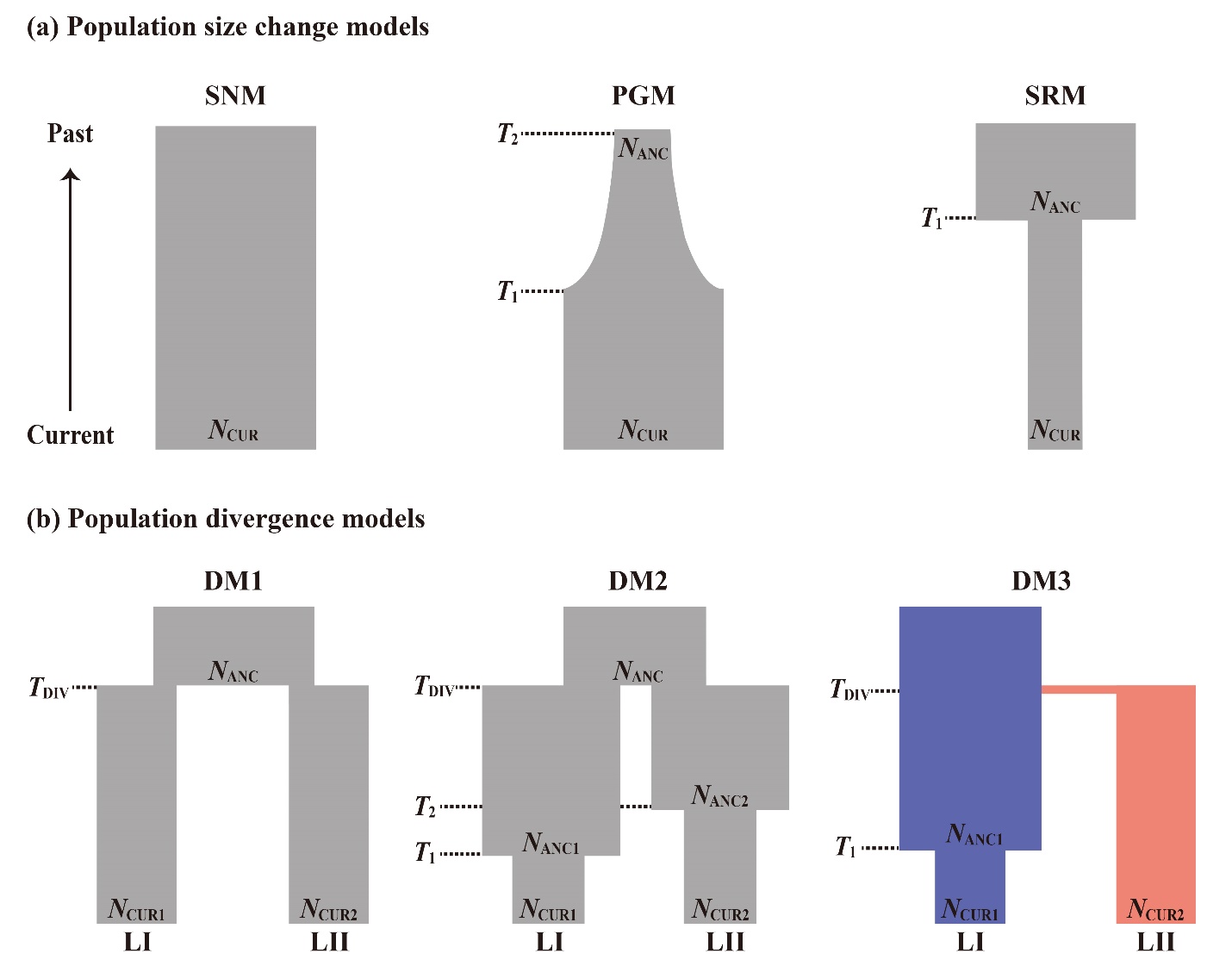
**

**Figure S4: Compared population size change and population divergence models (a and b, respectively)**. SNM, standard neutral model; PGM, population growth model; SRM, size reduction model; DM, divergence model. *N*_CUR_, current effective population size; *N*_ANC_, ancestral effective population size; *T*, event time for population size change; *T*_DIV_, divergence time.

**
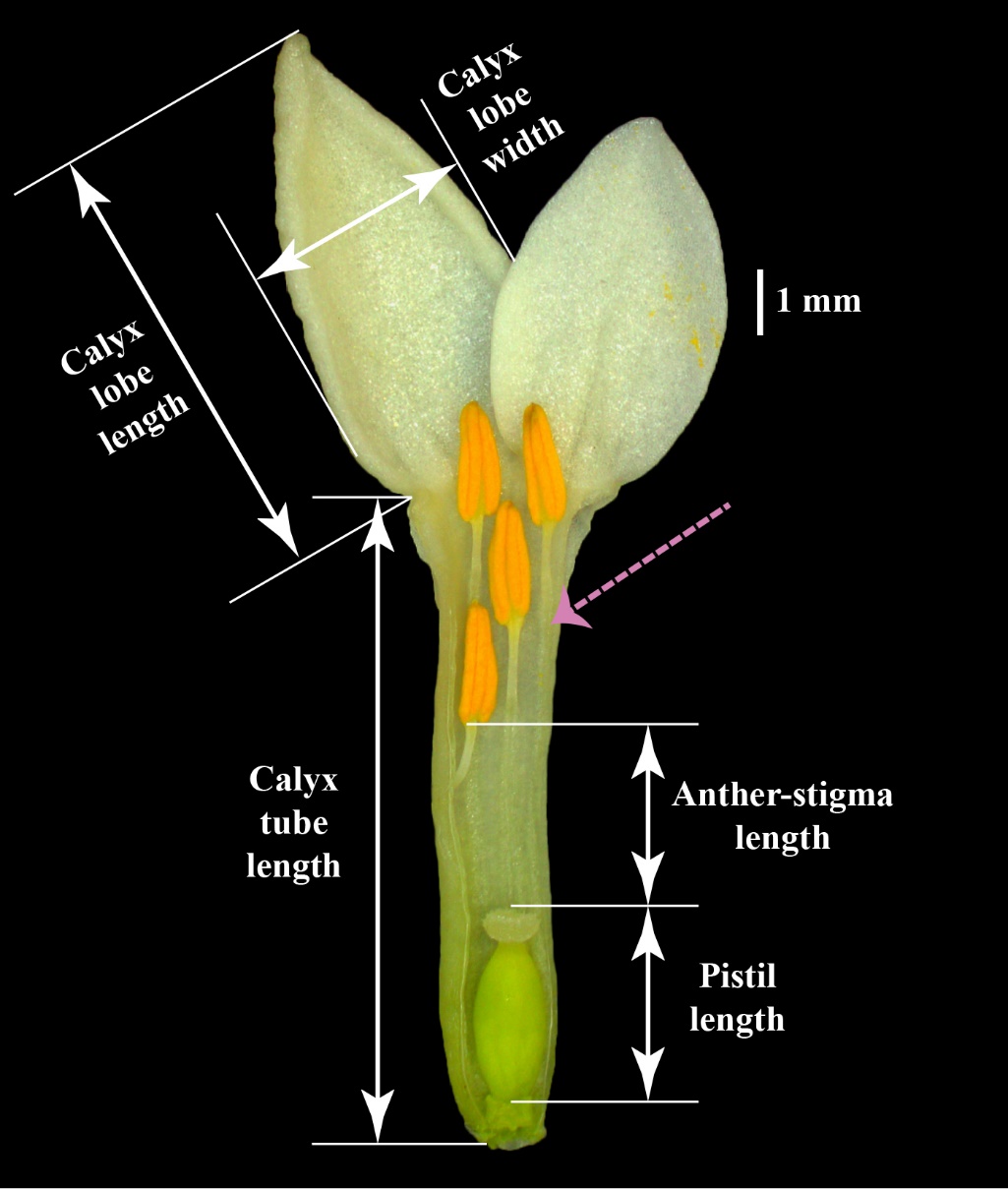
**

**Figure S5:**  **All floral measurements made to characterize variation in floral morphology.** The dashed arrow points to the fused filament of the stamen and calyx tube.

**
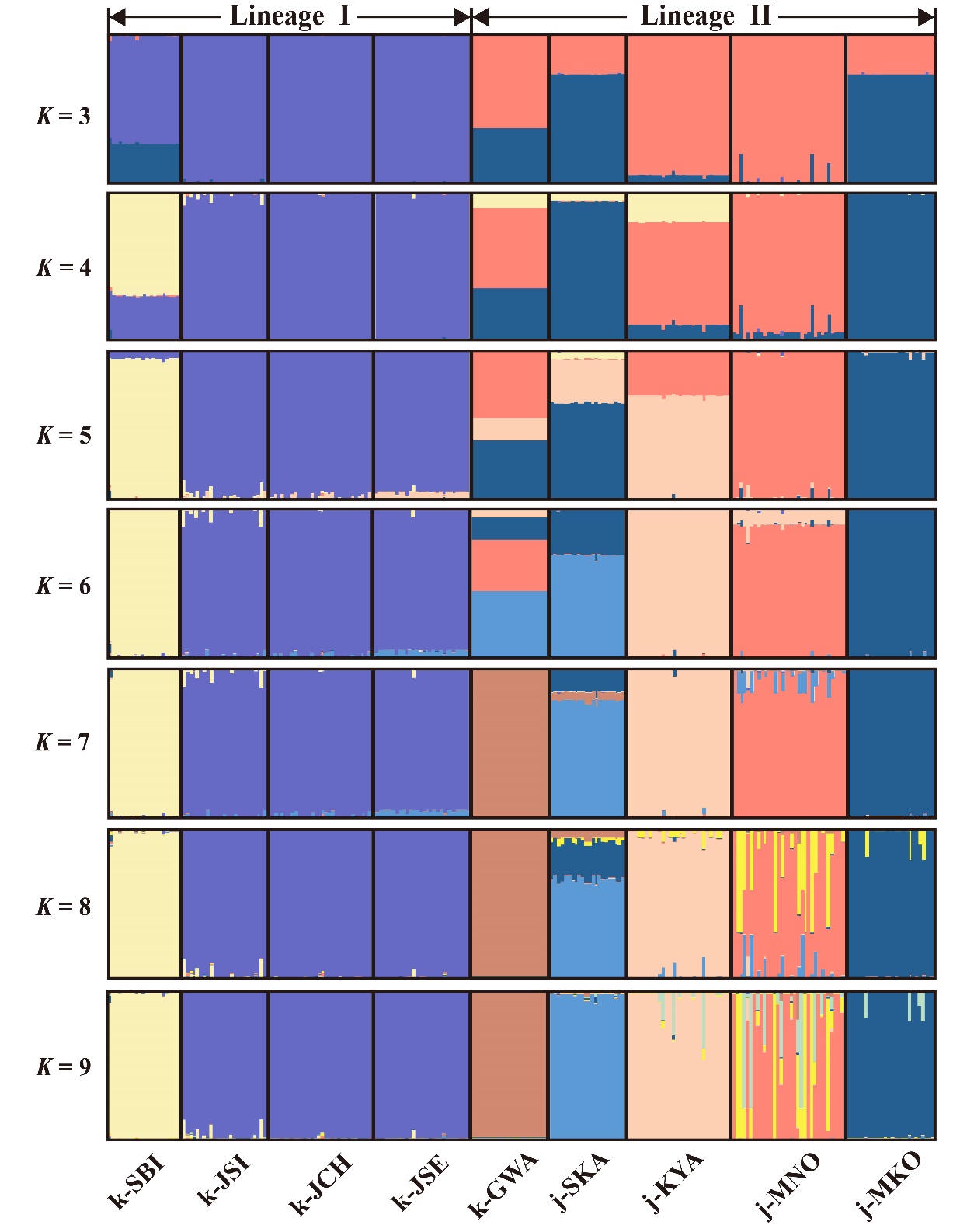
**

**Figure S6:** Bayesian individual-based clustering of *Daphne kiusiana* populations at *K* = 3 to *K* = 9. Each color depicts a cluster and each vertical bar represents an individual with the probability of membership to a cluster.

**
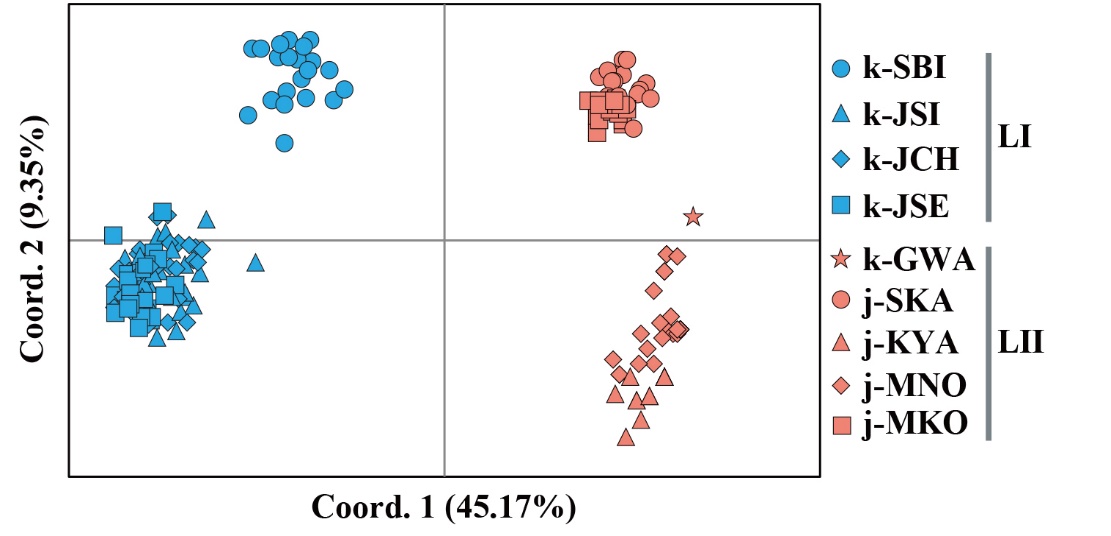
**

**Figure S7:**  **Principal coordinate analysis (PCoA) based on Nei’s genetic distance calculated from the allele frequencies of *Daphne kiusiana* 237 individuals.**

**
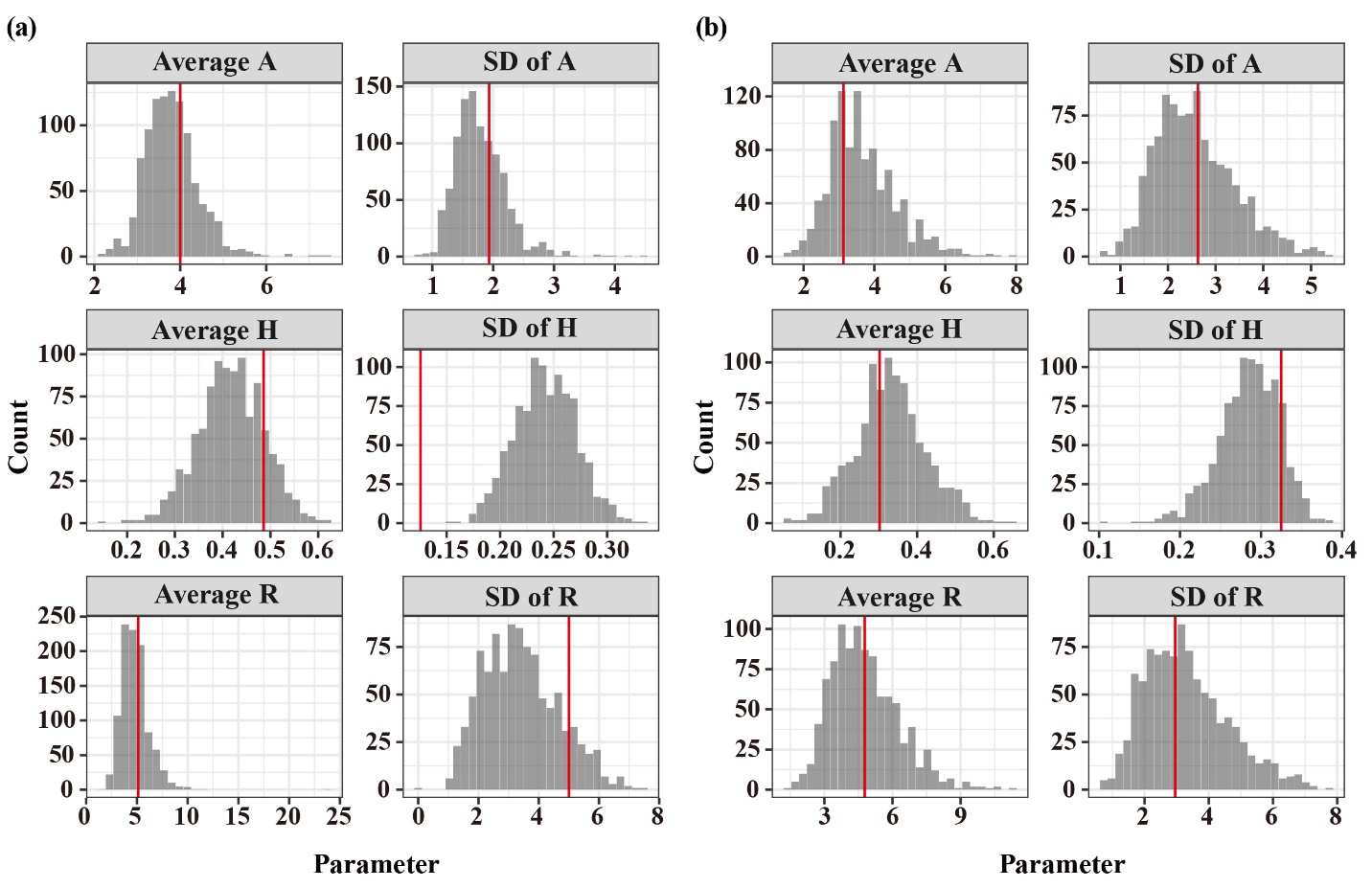
**

**Figure S8: Goodness-of-fit of the size reduction model (best model) for LI and LII (a and b, respectively).** The histograms and vertical bars indicate predicted and observed values, respectively. A, number of alleles; H, expected heterozygosity; R, allele size range.

**
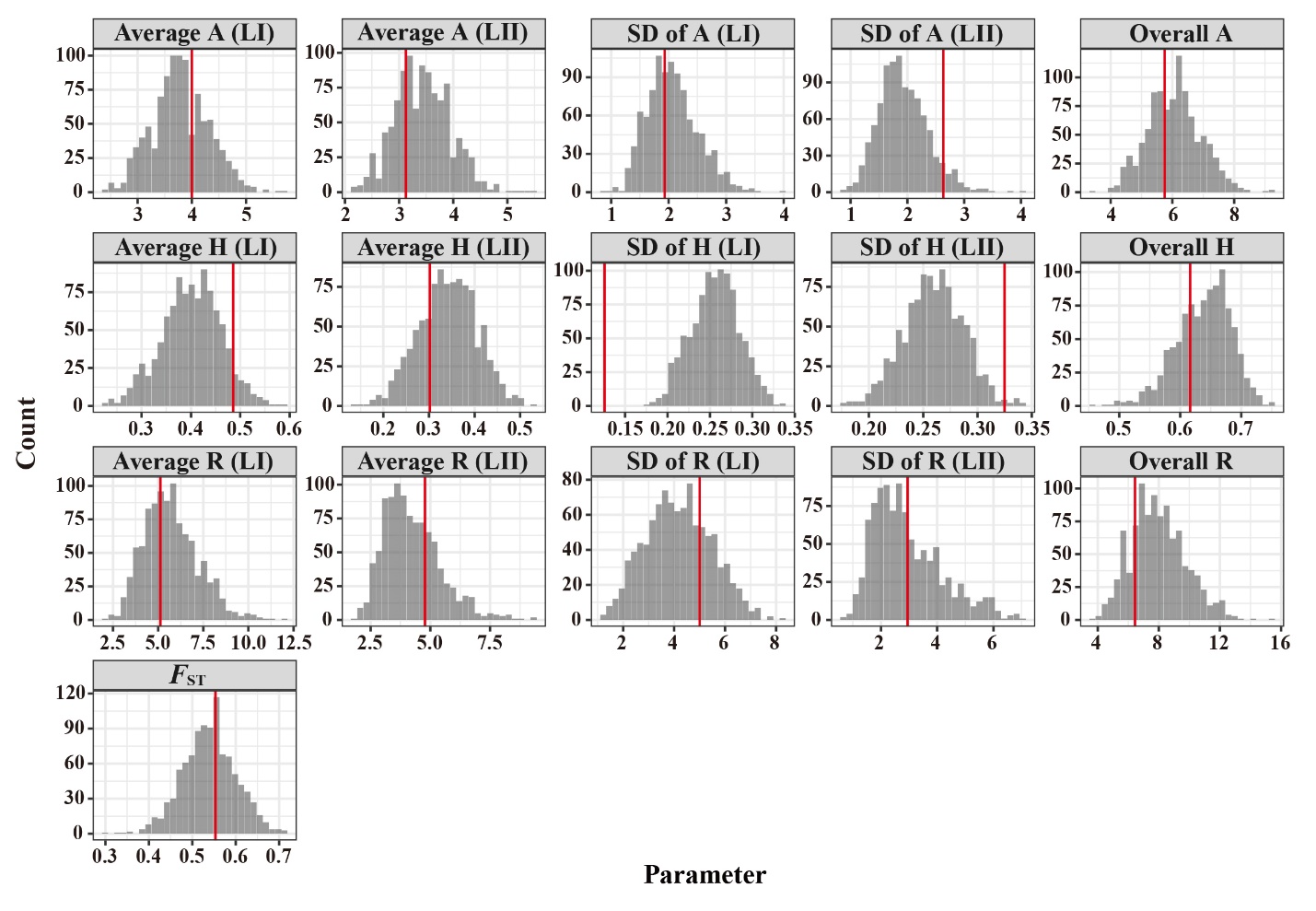
**

**Figure S9: Goodness-of-fit of the population divergence model 3 (best model).** The histograms and vertical bars indicate predicted and observed values, respectively. A, number of alleles; H, expected heterozygosity; R, allele size range.
