# Supplementary material for: Genetic and demographic signatures accompanying the evolution of the selfing syndrome in Daphne kiusiana, an evergreen shrub
Source: Ann Bot. 2022 Dec 5;131(5):751–67. doi: 10.1093/aob/mcac142 (PMC10184445; doi:10.1093/aob/mcac142)
Supplement: mcac142_suppl_Supplementary_Tables [file mcac142_suppl_supplementary_tables.docx]

**Table S1:** Developed 16 chloroplast DNA markers of *Daphne kiusiana* used in the analysis.

| Primer pair | Primer name | Primer sequences (5’→3’) | Region |
| --- | --- | --- | --- |
| *psbA*-*mat*K | *psb*A-F | GCGTCTCTCTAAAATTGCAG | LSC |
|  | *mat*K-R | GCGGGTTTGGTATTTGGATA |  |
| *mat*K-*trn*K(UUU)ex1 | *mat*K-F | GAGTTGTTGAAATTCCTCCA | LSC |
|  | *trn*K(UUU)-R | GGTAGAGTACTCGGCTTTTA |  |
| *trn*G-*atp*A | *trn*G-F | TATAACCCCTAGCCTTCCAA | LSC |
|  | *atp*A-R | GAAGCGGAAACACTTTTGAA |  |
| *rpo*C1 | *rpo*C1-F | TTTTAGTGCTGGGCCTAG | LSC |
|  | *rpo*C1-R | CTCTTAAAGAATTAGAGAGGCT |  |
| *trn*E-*trn*T | *trn*E-F | TGTCCTAAACCACTAGACGA | LSC |
|  | *trn*T-R | TACCACTGAGTTAAAAGGGC |  |
| *psb*C-*psb*Z | *psb*C-F | ACCTGTTCTTTCGATGACTC | LSC |
|  | *psb*Z-R | CGGCCAATTGGAAAGCTATA |  |
| *ycf*1-*ycf*2 | *ycf*ex2-F | TAATTGCCTTCGGATTGAGC | LSC |
|  | *ycf*ex1-R | TTACAGAGATGGTGCGATTT |  |
| *trn*L-*trn*F | *trn*L-F | GGGATATGGCGAAATTGGTA | LSC |
|  | *trn*F-R | CTCTACCAACTGAGCTATCC |  |
| *trn*V-*trn*M | *trn*V-F | CTCGAACCGTAGACTTTCTC | LSC |
|  | *trn*M*-*R | AGCAATACTCTAACCACTGA |  |
| *atp*B-*rbc*L | *atp*B-F | AGGATCTGAAGTAGCAGGAT | LSC |
|  | *rbc*L-R | TAGTCTCTGTTTGTGGTGAC |  |
| *acc*D-*psa*I | *acc*D-F | TGACTCCAACTCCAAATCAG | LSC |
|  | *psa*I-R | AAATGGAGGGTAAGCTGTTG |  |
| *ycf*4-*cem*A | *ycf*4-F | TGATGAAAATTTGACTCCGC | LSC |
|  | *cem*A-R | TTGAATGAGAGCGAAATCCA |  |
| *pet*A-*psb*J | *pet*A-F | AAACAGTTTGAGAAGGTTCA | LSC |
|  | *psb*J-R | ATTCTGGATTGGGTTCATCC |  |
| *pet*L-*trn*P | *pet*L-F | TGGTCTGAGTAAGATACGAC | LSC |
|  | *trn*P-R | GTTCAAATCCTGTCATCCCT |  |
| *rpl*11-*rps*8 | *rpl*11-F | TTGGTATTCTACGTGCATCC | LSC |
|  | *rps*8-R | GGCGGAGAAATTTTGTGTTA |  |
| *ndh*F-*rpl*32 | *ndh*F-F | CCCAAGACCATACATATTGATA | SSC |
|  | *rpl*32-R | CCTGTAGAAAGAGATTTTGCTA |  |

**Table S2:** Prior distribution of compared population size change and population divergence models.

| Model | Parameter | Prior distribution |
| --- | --- | --- |
| SNM and PGM | *N*_CUR_ | Uniform (2×10^3^, 2×10^4^) |
| SRM | *N*_CUR_ | Uniform (10, 2×10^3^) |
| PGM | *N*_ANC_ | Uniform (10, 2×10^3^) |
| SRM | *N*_ANC_ | Uniform (2×10^3^, 2×10^4^) |
| PGM and SRM | *T*_1_ | Log uniform (10^0^, 10^5^) |
| PGM | *T_2_*^a^ | Log uniform (10^0^, 10^5^) |
|  |  |  |
| DM1, DM2 and DM3 | *N*_CUR_1^b^ | Fixed into 468 |
| DM1, DM2 and DM3 | *N*_CUR_2^b^ | Fixed into 439 |
| DM2 and DM3 | *N*_ANC_1^b^ | Fixed into 10354 |
| DM2 | *N*_ANC_2^b^ | Fixed into 3364 |
| DM1 and DM2 | *N*_ANC_ | Uniform (2×10^3^, 5×10^4^) |
| DM2 and DM3 | *T*1^‡^ | Fixed into 1206 |
| DM2 | *T*2^‡^ | Fixed into 5405 |
| DM1 | *T*_DIV_ | Log uniform (10^0^, 10^6^) |
| DM2 | *T*_DIV_ | Log uniform (5405, 10^6^) |
| DM3 | *T*_DIV_ | Log uniform (1206, 10^6^) |
|  |  |  |
| All | *shape* | Uniform (0.5, 5) |
| All | *P*_GSM_ | Uniform (0, 1) |

^a^ T_1_ < T_2_.

^b^ Posterior mode values estimated in the single population size change analysis were used.

Notes: The units of the effective population size and time parameters are the number of diploid individuals and generations ago, respectively. SNM, standard neutral model; PGM, population growth model; SRM, size reduction model; DM, divergence model. *N*_CUR_, current effective population size; *N*_ANC_, ancestral effective population size; *T*, event time for population size change; *T*_DIV_, divergence time; shape and *P*_GSM_, generalized stepwise mutation model parameters.
